# Fabricating 2D MoS_2_ with Edge Sulfur Vacancy Defects by Heavy Ion Bombardment Shear‐Exfoliation for Enhanced Sodium Storage

**DOI:** 10.1002/advs.202417576

**Published:** 2025-07-15

**Authors:** Feiyan Mu, Siqi Li, Dongqi Zhang, Qing Zhang, Zhe Hu, Ye Han, Zhixin Tai, Yajie Liu

**Affiliations:** ^1^ Advanced Energy Storage Materials and Technology Research Center Guangdong‐Hong Kong Joint Laboratory for Carbon Neutrality Jiangmen Laboratory of Carbon Science and Technology Jiangmen 529199 China; ^2^ Shenzhen Key Laboratory of Advanced Energy Storage Department of Mechanical and Energy Engineering Southern University of Science and Technology Shenzhen 518055 China; ^3^ Guangdong Provincial Key Laboratory of Service Safety for New Energy Materials College of Materials Science and Engineering Shenzhen University Shenzhen 518055 China; ^4^ School of Science Changchun University Changchun 130022 China

**Keywords:** metal ion assisted, S‐vacancies, shear exfoliation, sodium‐ion batteries

## Abstract

Vacancy engineering is widely considered an effective approach to modulate the internal electronic structure of electrode materials, enhancing charge‐transfer processes/reactions and leading to excellent energy storage properties. Nevertheless, several current techniques of vacancy engineering, such as controlled solvent thermal growth, plasma bombardment, and chemical etching, suffer from high energy inputs and uncontrollable processing kinetics. Herein, a facile and energy‐efficient technique of metal ion‐assisted shear exfoliation is proposed to synthesize 2D MoS_2_ with edge S‐vacancies as an anode for sodium ion batteries. Thanks to the implementation of this vacancy technique, few‐layer MoS_2_ anode with sulfur defects at the edge presents remarkable rate performance (399.91 mAh g^−1^ at a current density of 5 A g^−1^) and demonstrates high average capacity with exceptional stability (460.71 mAh g^−1^ at 1 A g^−1^ after 100 cycles) when utilized in sodium‐ion batteries. The superior electrochemical performance of this elaborate anode can be ascribed to the enhanced electrochemical kinetics and reaction reversibility resulting from the presence of a vacancy defect architecture. This study is expected to provide an effective avenue to develop vacancy defect electrodes for advanced batteries.

## Graphical abstract

Few‐layered MoS_2_ with edge sulfur vacancies (L‐MoS_2‐x_) was prepared via heavy ion bombardment shear‐exfoliation. This anode demonstrates superior sodium storage performance, delivering 399.91 mAh g^−1^ at 5 A g^−1^ and maintaining 460.71 mAh g^−1^ at 1 A g^−1^ after 100 cycles in sodium‐ion batteries (SIBs). 

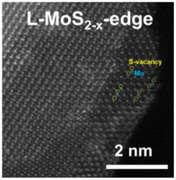

## 1 Introduction

The layered transition metal disulfides (TMDs), MS_2_ (M = Mo, W, V, and Ti), are considered highly desirable anodes for sodium storage in view of their large interlayer space and relatively high theoretical capacity. However, the low conductivity, limited active sites, and insufficient charge/mass transfer significantly compromise the performance of Sodium‐Ion Batteries (SIBs).^[^ ^1^, ^2^, ^3^, ^4^ ^]^ Vacancy engineering is widely acknowledged as an effective strategy for addressing aforementioned issues and fabricating advanced TMDs anode materials due to its advantages of adjusting the electronic structure, optimizing the conductivity, and enhancing the charge‐transfer processes.^[^ ^5^ ^]^ So far, various techniques of vacancy engineering of layered transition metal disulfides have been extensively explored and reported to tackle the problems. As Hu and co‐workers reported, the few‐layer MoS_2_ nanosheets with S‐vacancies were immobilized onto 3D flower‐doped N‐carbon frameworks (NCF@V‐MoS_2_) by a hydrothermal method and chemical etching strategy. The introduction of S‐vacancies on the surface of MoS_2_ nanosheets not only tunes the electronic structure and essentially improves the electrical conductivity but also expands the interlayer distance and accelerates the Na^+^ diffusion. Benefiting from the synergistic effect of conductive NCF and V‐MoS_2_, the synthesized NCF@V‐MoS_2_ presents enhanced specific capacity and cycling stability in SIBs.^[^ ^6^ ^]^ Dong et al. synthesized a series of co‐doping vanadium tetrasulfide/reduced graphene oxide (rGO) composites, exhibiting a 2D ultrathin layered structure, as the active sulfur host material in Li‐S batteries. Besides Co doping, S defects in VS_4_ disrupted the regular arrangement of the local lattice, which enhanced the electrocatalytic activity, greatly accelerated the formation of Li_2_S, and thereby significantly improved the electrochemical performance of the battery.^[^ ^7^ ^]^ In addition to the above chemical etching, various techniques such as heteroatom doping to create vacancies, coupled with plasma bombardment,^[^ ^8^ ^]^ controlled solvothermal growth,^[^ ^9^ ^]^ and other vacancy engineering techniques have been extensively developed to construct S vacancy in TMDs and the introduction of S vacancy significantly enhances their electrochemical performance. Zheng et al. employed a hydrothermal method in combination with carbothermal reduction and vulcanization to induce abundant sulfur vacancies within CoS_2_/FeS_2_ heterojunctions, thereby promoting sodium ion adoption and transport.^[^ ^10^ ^]^ Despite the availability of various preparation techniques for introducing sulfur vacancies, conventional strategies, nevertheless, exhibit limitations in terms of high energy input and uncontrollable processing kinetics,^[^ ^11^ ^]^ thereby restricting their application in large‐scale production of electrode materials with vacancy defects.^[^ ^12^ ^]^ The development of a controllable, feasible, and cost‐effective preparation technique for vacancy engineering is therefore indispensable and of great importance.

High‐shear exfoliation represents a cost‐effective liquid‐phase exfoliation (LPE) method suitable for large‐scale production of defect‐free few‐layer 2D materials. Optimizing exfoliation conditions and solvents during the preparation process is a common strategy to enhance yields of defect‐free 2D materials. As an initial example, Keith R. Paton et al. developed an industrially scalable high‐speed shear exfoliation technique for the production of large quantities of defect‐free few‐layer graphene in the N‐methyl‐2‐pyrrolidone (NMP)‐surfactant solution.^[^ ^13^ ^]^ Subsequently, our research group conducted a series of investigations and advancements in the shear‐assisted mechanical exfoliation technique.^[^ ^14^ ^]^ For instance, Hu et al. employed high shear exfoliation to synthesize ultrathin titanium sulfide nanosheets, and the cells based on these nanosheets demonstrated excellent cycling performance (386 mAh g^−1^ after 200 cycles at 0.2 A g^−1^).^[^ ^14c^ ^]^ Tai et al. expanded the strategy of shear‐exfoliation to layered cathode materials and demonstrated that few‐layer structured transition metal oxide cathodes are able to render excellent C‐rate capability and long‐term cycling performance, while experiencing significantly reduced volume expansion during cycling. Moreover, in addition to developing mixture exfoliation solvents for few‐layer Sb_2_S_3_ anode in advanced potassium ion batteries by Liu et al., they also made a groundbreaking discovery of the phenomenon of organic solvents in situ carbonization during shear exfoliation, which opens a new avenue toward carbon composite fabrication with selected organic solvents.^[^ ^14b^ ^]^ Until now, the majority of reports on shear exfoliation have primarily focused on achieving high‐yield defect‐free 2D material for various applications through mechanical exfoliation. However, there has been limited research dedicated to investigating defect generation, amplification, and their potential applications by shear exfoliation. Therefore, in this work, we lead pioneer research on vacancy regulation through shear exfoliation and achieve a breakthrough in utilizing shear exfoliation for vacancy engineering of 2D materials.

Herein, few‐layered MoS_2_ with edge sulfur vacancy (L‐MoS_2‐x_) is prepared via an effective, easily scaled‐up, two‐step heavy ion bombardment shear‐exfoliation technique, aiming to boost the electrochemical performance for sodium storage. The fatal issue of huge volume changes and kinetic degradation during electrochemical cycling can be solved by a few‐layer structured design of MoS_2_, which is fabricated via solution‐triggered first‐step shear exfoliation as previously proposed by us.^[^ ^14^, ^15^ ^]^ Furthermore, L‐MoS_2‐x_ with edge S vacancy is fabricated by a second‐step metal ion‐assisted shear exfoliation technique, which is triggered by S‐vacancy lattice defects. These edge S‐vacancies can dramatically facilitate the transport/transfer of Na^+^, thus promoting electrochemical kinetics and reaction reversibility effectively. By harnessing the synergistic effect between a few‐layer structure and S vacancy defects, cells with L‐MoS_2‐x_ anodes perform excellent rate capability and long‐term stability.

## 2 Results and Discussion

### 2.1 Preparation and Characterization of S‐Vacancies

**Figure 1:**
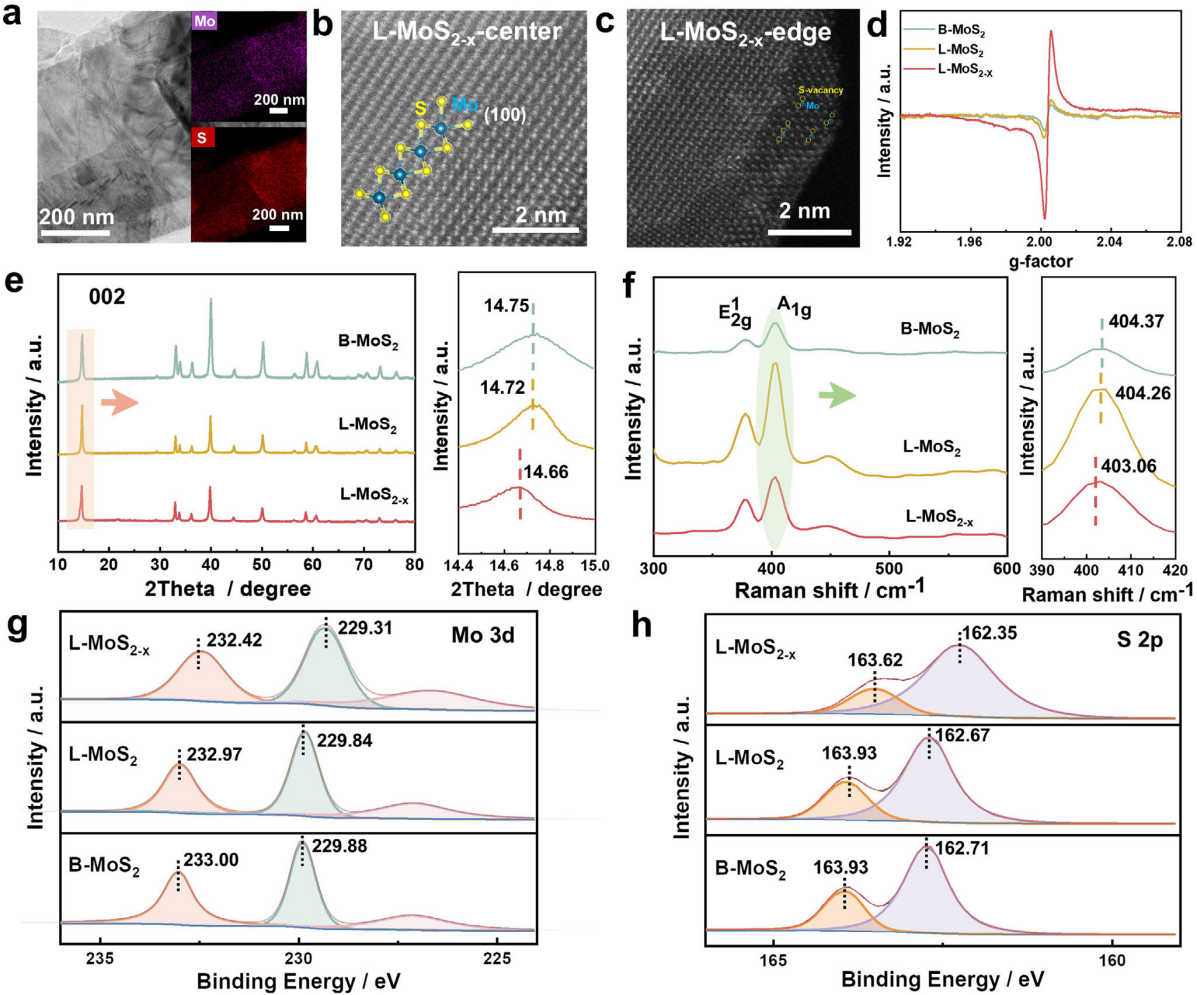
Characterization of S vacancies. a) High‐resolution TEM (HRTEM) image of L‐MoS_2_. b, c) Spherical aberration‐corrected scanning transmission electron microscopy (AC‐TEM) of the L‐MoS_2‐x_. d) Electron paramagnetic resonance (EPR) characterizations of MoS_2_, L‐MoS_2_, and L‐MoS_2‐x_. e)X‐ray diffraction (XRD) patterns of MoS_2_, L‐MoS_2_, and L‐MoS_2‐x_. f) Raman spectra of B‐MoS_2_, L‐MoS_2_, and L‐MoS_2‐x_. g) High‐resolution Mo 3d spectrum of MoS_2_, L‐MoS_2_ and L‐MoS_2‐x_. h) High‐resolution S 2p spectrum of MoS_2_, L‐MoS_2_ and L‐MoS_2‐x_.

The few‐layered MoS_2_ with edge sulfur vacancy (L‐MoS_2‐x_) was prepared through a two‐step high shear exfoliation process. The bulk Molybdenum disulfide (B‐MoS_2_) was exfoliated into a few layers of MoS_2_ (L‐MoS_2_) during the first exfoliation, utilizing a mixture of water and ethanol as solvent. The introduction of vacancy defects in L‐MoS_2_ was made through the second exfoliation process with cobalt salt as an additive in the exfoliation solvents, resulting in the formation of L‐MoS_2‐x_ (Figure S1, Supporting Information). The morphology and particle distribution of the as‐prepared exfoliated products were investigated by scanning electron microscope (SEM) and high‐resolution transmission electron microscopy (HRTEM). These measurements reveal a few‐layered laminar morphology for L‐MoS_2_ and L‐MoS_2‐x_, while B‐MoS_2_ exhibits an aggregated bulk morphology (**Figure** **1**a; Figures S2 and S3, Supporting Information), indicating the successful mechanical exfoliation of B‐MoS_2_. In order to visually observe the S vacancies in L‐MoS_2‐x_ prepared via metal‐assisted shear exfoliation, we conducted spherical aberration‐corrected scanning transmission electron microscopy (AC‐TEM) analysis, and the corresponding results are shown in Figure 1b,c. The images of L‐MoS_2‐x_ reveal that the presence of normal S atom spots at the center of the material, while the lack of such spots on the edges, thereby confirming the existence of the S‐vacancies along the edges. In contrast, both B‐MoS_2_ and L‐MoS_2_ exhibit a negligible presence of vacancy defects in the center as well as at the edge (Figure S4, Supporting Information), suggesting that the heavy metal ions‐containing solvents play an important role in shear exfoliation. To further confirm the presence of S vacancies in MoS_2_, we carried out electron paramagnetic resonance (EPR) analysis on B‐MoS_2_, L‐MoS_2_, and L‐MoS_2‐x_. The characteristic peak of the Mo‐S bond at g = 2.009 can be clearly observed in Figure 1d. The significantly augmented intensity of L‐MoS_2‐x_ in comparison to MoS_2_ and L‐MoS_2_ further corroborates the presence of abundant S‐vacancies in L‐MoS_2‐x_, as the intensity of this peak is proportional to the concentration of S‐vacancies in MoS_2_.

The X‐ray diffraction (XRD) results in Figure 1e demonstrate that the diffraction peaks are in good agreement with the standard 2H‐MoS_2_ (PDF#37‐1492), which indicates that both the L‐MoS_2_ and L‐MoS_2‐x_ maintain a favorable crystal structure. Compared to the B‐MoS_2_, the (002) peaks of L‐MoS_2_ and L‐MoS_2‐x_ exhibit a shift toward a lower angle in the magnified XRD patterns image, suggesting an expanded interlayer spacing and weakened van der Waals interactions. The Raman spectra of B‐MoS_2_, L‐MoS_2_, and L‐MoS_2‐x_ are illustrated in Figure 1f. The two apparent peaks observed at ≈380 and 405 cm^−1^ correspond to the in‐plane $E_{2g}^{1}$ and out‐of‐plane A_1g_ vibrational modes of MoS_2_, respectively. The sequential reduction in the distance between the $E_{2g}^{1}$ peak and the A_1g_ peak among of B‐MoS_2_, L‐MoS_2_, and L‐MoS_2‐x_, indicates a decreased strength of Mo‐S chemical bonding. A smaller distance was observed between the $E_{2g}^{1}\;$ peak and the A_1g_ A peak in L‐MoS_2‐x_ compared to that in L‐MoS_2_ may result from S‐vacancy implantation within L‐MoS_2‐x_. Furthermore, X‐ray photoelectron spectroscopy (XPS) measurements were performed to investigate the chemical composition and valence state of the as‐prepared samples. In the high‐resolution XPS spectrum of Mo 3d shown in Figure 1g and Figure S5 (Supporting Information), the peaks observed at 229.88 and 233.00 eV, can be ascribed to Mo 3d 5/2 and Mo 3d 3/2, respectively, while the signal at 227.03 eV is assigned to S 2s. In the S 2p spectrum (Figure 1h), in contrast to both B‐MoS_2_ and L‐MoS_2_, the main peaks in L‐MoS_2‐x_ obviously shift toward low binding energies, indicating an enhanced electron density around the *S* sites resulting from the formation of *S* vacancies during metal ion‐assisted shear exfoliation. The potential underlying mechanism of heavy ion‐assisted shear exfoliation was investigated via density functional theory (DFT) calculation. As shown in Figure S6 (Supporting Information), the Mo‐S bond length in MoS_2_ increases from 2.41 to 2.46 upon the interaction with Co ions, illustrating that an appropriate heavy ions environment in solvents can weaken the bonding strength between Mo and S. Furthermore, during the exfoliation process, the shear force locally stretches Mo‐S bonds, enabling Co^2+^ to selectively attack the weakened *S* sites and facilitating the formation of *S*‐vacancies under the high‐speed shear exfoliation (Figures S7 and S8, Supporting Information).

### 2.2 Electrochemical Performance of Various MoS_2_‐Based Materials

To investigate the impact of exfoliation duration on electrochemical performance, we conducted the electrochemical tests on MoS_2_‐based anode materials prepared under different exfoliation durations (Figures S9 and S10, Supporting Information). The corresponding results ultimately suggested that the electrode stability during cycling can be effectively maintained by employing an appropriate layered structure of MoS_2_ through 2 h shear‐exfoliation. This optimal exfoliation time at 6000 rpm is 2 h in the first‐step exfoliation, which will also be used for subsequent samples. To investigate the impact of Co(NO_3_)_2_ as an solvent additive on the electrochemical performance of L‐MoS_2_ electrodes, we compared the cycling stability and rate capability of the different electrodes (L‐MoS_2‐x_‐1, L‐MoS_2‐x_‐2, L‐MoS_2‐x_‐3, L‐MoS_2‐x_‐4 indicating 10, 30, 50, and 70 g L^−1^ of Co(NO_3_)_2_ concentration in exfoliation solvents, respectively) (Figures S11 and S12, Supporting Information). The presence of salt additives in exfoliation solvents has been found to promote the formation of *S* vacancy defects in Figure 1 and Figure S13 (Supporting Information). In comparison to the L‐MoS_2_ sample, the counterpart samples prepared via metal‐assisted exfoliation with S defects present enhanced capacity and improved cycling stability. Meanwhile, according to the aforementioned electrochemical results, the optimal concentration of Co(NO_3_)_2_ in the solvent was determined to be 50 g L^−1^. Consequently, subsequent analysis of L‐MoS_2‐x_ will be conducted using this optimized solvent additive concentration.

**Figure 2:**
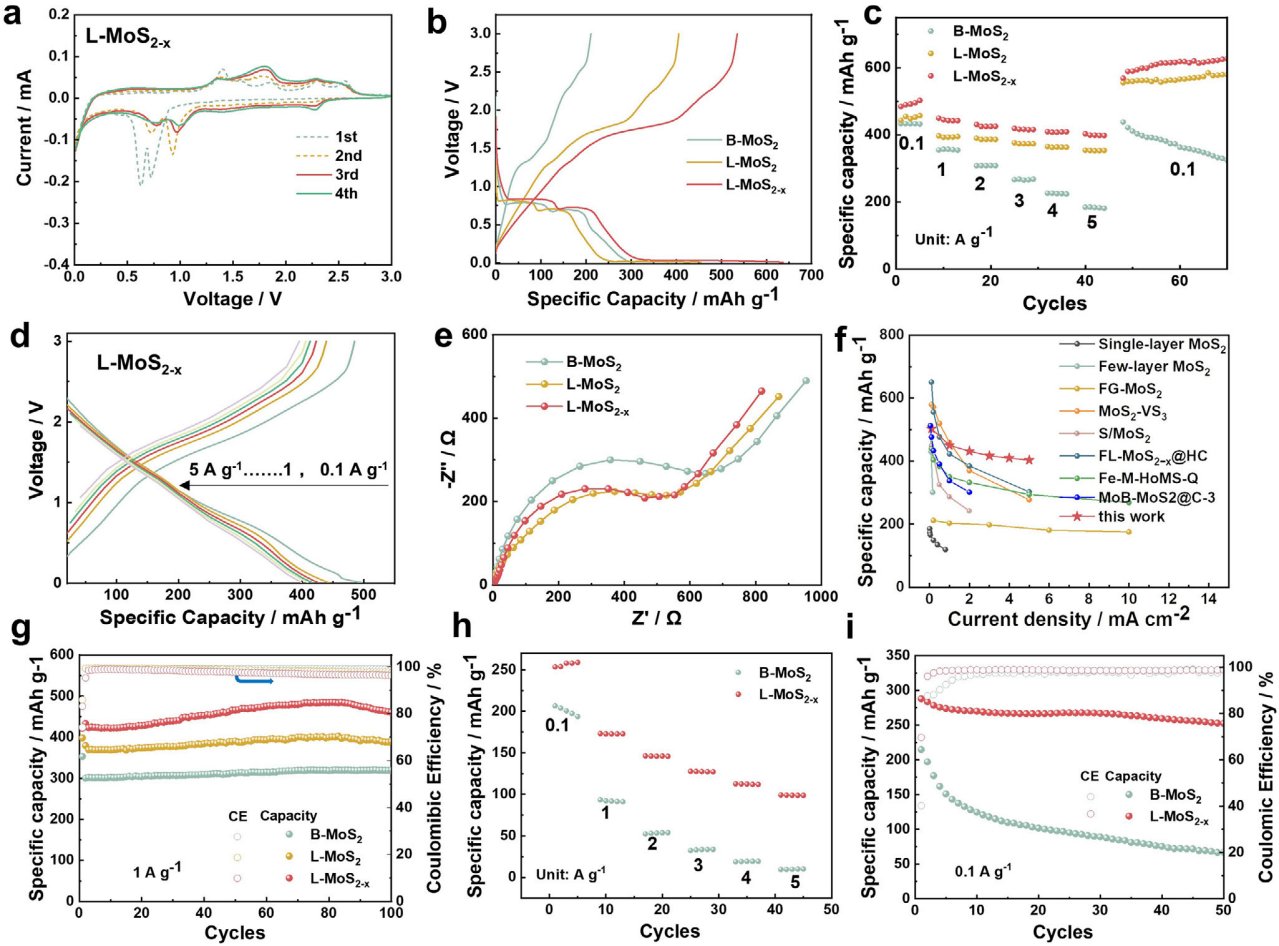
Electrochemical performance of materials. a) CV curves of L‐MoS_2‐x_ at 0.1 mV s^−1^. b) The initial galvanostatic charge‐discharge curves of B‐MoS_2_, L‐MoS_2_ and L‐MoS_2‐x_ at 0.1 A g^−1^ in the voltage range of 0.01–3.0 V. c) Rate performances of B‐MoS_2_, L‐MoS_2_, and L‐MoS_2‐x_. d) Charge‐discharge profiles of L‐MoS_2‐x_ at different rates. e) Nyquist plot of electrochemical impedance spectra of B‐MoS_2_, L‐MoS_2_, and L‐MoS_2‐x_. f) Comparison of the Na^+^ storage performance of the L‐MoS_2‐x_ in this work with previously reported MoS_2_‐based materials.^[^ ^12^, ^18^ ^]^ g) Cycling performances of MoS_2_, L‐MoS_2_ and L‐MoS_2‐x_ at 1 A g^−1^. h) Rate performance of B‐MoS_2_//NMF111 and L‐MoS_2‐x_//NMF111 full cells at different current densities. i) Cycling performances of B‐MoS_2_//NMF111 and L‐MoS_2‐x_//NMF111 full cells at current density of 0.1 A g^−1^.

Electrochemical properties of the layer‐structured L‐MoS_2_ and defect‐engineered L‐MoS_2‐x_ anodes were investigated within the range of 0.01–3 V (vs Na^+^/Na). **Figure** **2**a and Figure S14 (Supporting Information) depict typical cyclic voltammograms of the MoS_2_‐based electrodes during the initial four cycles at a scan rate of 0.1 mV s^−1^. The L‐MoS_2‐x_ electrode exhibits two reduction peaks at ≈0.72 and 0.63 V, respectively, during the first cycle cathodic scan. The peaks can be attributed to the intercalation of Na^+^ ions and the formation of solid electrolyte interface (SEI) films, as well as the conversion reaction from Na_x_MoS_2_ to Na_2_S/Mo. The oxidation peaks observed at ≈1.4 and 1.8 V are attributed to the reverse reaction of metallic Mo nano‐grains and NaS_2_, whereas a small anodic peak at 2.3 V represents the partial oxidation of Na_2_S to S or polysulfide.^[^ ^16^ ^]^ The subsequent CV curves of the L‐MoS_2‐x_ electrodes overlap satisfactorily after the second cycle, indicating good electrochemical reversibility and stability. The specific reaction mechanism and structural evolution were further investigated via in‐situ XRD of different electrodes (Figure S15, Supporting Information), and the results indicate that the layered and defective material of L‐MoS_2‐x_ can enhance electrochemical reaction kinetics and facilitate the reversible progression of the redox reaction during discharge/charge. The galvanostatic charge‐discharge (GCD) curves of B‐MoS_2_, L‐MoS_2_, and L‐MoS_2‐x_ for the initial cycle at the current density of 0.1 A g^−1^ are displayed in Figure 2b. The L‐MoS_2‐x_ electrode exhibited the highest discharge and charging specific capacity (703.45 and 598.37 mAh g^−1^, respectively) among the tested electrodes, suggesting that the layer‐structured MoS_2_ with S vacancies could provide more abundant active sites for Na^+^ storage. Although the initial coulombic efficiency (ICE) of L‐MoS_2‐x_ (ICE = 85.06%) is relatively lower than that of L‐MoS_2_ (ICE = 89.2%) attributed to the partial irreversibility caused by Na^+^ ions trapping and side reactions from electrolyte decomposition, it remains comparable to that of L‐MoS_2_ in terms of reversible capacity. The rate capability of three electrodes was evaluated at different current densities as depicted in Figure 2c. Among them, the S‐vacancies electrode demonstrated superior rate capability compared to MoS_2_ and L‐MoS_2_. The average discharge capacities at current densities of 0.1, 1, 2, 3, 4, and 5 A g^−1^, respectively, were 492.93, 444.72, 426.6, 416.92, 408.79, and 399.91 mAh g^−1^, respectively. The capacity of B‐MoS_2_ decreases upon reverting to 0.1 A g^−1^, while electrodes L‐MoS_2_ and L‐MoS_2‐x_ can maintain or even increase the capacity retention, suggesting the structural integrity, increased active sites and reversible expansion of the (002) interplanar spacing in few‐layer structured MoS_2_ induced by rapid Na^+^ insertion/extraction during high‐rate cycling (Figure S16, Supporting Information).^[^ ^17^ ^]^ For the S‐vacancy electrode, the rate performance and the corresponding charge/discharge profiles at different rates (Figure 2d; Figures S17 and S18, Supporting Information) substantiate that the introduction of *S* vacancies could contribute to the fast ion transfer and improved reaction kinetics. Additionally, the enhanced charge transfer process is further confirmed by the low charge transfer resistance observed in the L‐MoS_2‐x_ based cell obtained from electrochemical impedance spectra (Figure 2e). Surprisingly, the MoS_2_ with S‐vacancies obtained through shear‐exfoliation exhibit a significantly enhanced rate capability compared to previously reported state‐of‐the‐art anodes based on MoS_2_, demonstrating its great potential and promising application in fast charge‐discharge energy storage devices (Figure 2f). The cycling properties at different current densities were also investigated to ascertain the stability of MoS_2_‐based electrodes (Figure 2g; Figure S19, Supporting Information). The L‐MoS_2‐x_ presents a reversible capacity of 636.02 mAh g^−1^ at 0.1 A g^−1^ with an approximate CE of 100%, while maintaining a capacity of ≈594.11 mAh g^−1^ after 100 cycles. While the L‐MoS_2_ and B‐MoS_2_ electrodes exhibit a low reversible capacity of 457.15 and 186.43 mAh g^−1^, respectively, after 100 cycles, indicating poor cycling performance and limited capacity retention. The cycling stability of the L‐MoS_2‐x_ electrode was further assessed at a high current density of 1 A g^−1^ (Figure 2g), demonstrating a reversible capacity of 460.71 mAh g^−1^ over 100 cycles, surpassing that of both B‐MoS_2_ and L‐MoS_2_ electrodes. In order to assess the vacancy retention and structural evaluation during cycling, aberration‐corrected transmission electron microscopy (AC‐TEM) and XPS were conducted (Figure S20, Supporting Information) and the results confirm that the the structure of L‐MoS_2‐x_ with sulfur vacancies can be retained during cycling and these vacancies architecture may provide more active sites and facilitate the ion transport during cycling. To further assess the potential practical application of L‐MoS_2‐x_, we constructed a sodium‐ion full cell by using L‐MoS_2‐x_ as the anode and NaNi_1/3_Fe_1/3_Mn_1/3_O_2_ (NFM111) as the cathode. The galvanostatic charging/discharging profiles of such a full cell for the first three cycles are presented in Figure S21 (Supporting Information), indicative of its high stability. The rate capabilities of full cells were performed under various current densities (Figure 2h). As the current density increases from 0.1 to 2 A g^−1^, the L‐MoS_2‐x_//NFM111 full cell exhibits the reversible capacities of ≈228 and ≈146 mAh g^−1^, compared to those of B‐MoS_2_//NFM111. The L‐MoS_2‐x_//NFM111 electrode exhibits a remarkable specific capacity of 252.45 mAh g^−1^ after 50 cycles at a current density of 0.1 A g^−1^, accompanied by an impressive capacity retention of ≈88.9%.

### 2.3 S‐Vacancies Promote Na^+^ Reaction Kinetics and Transport Kinetics

**Figure 3:**
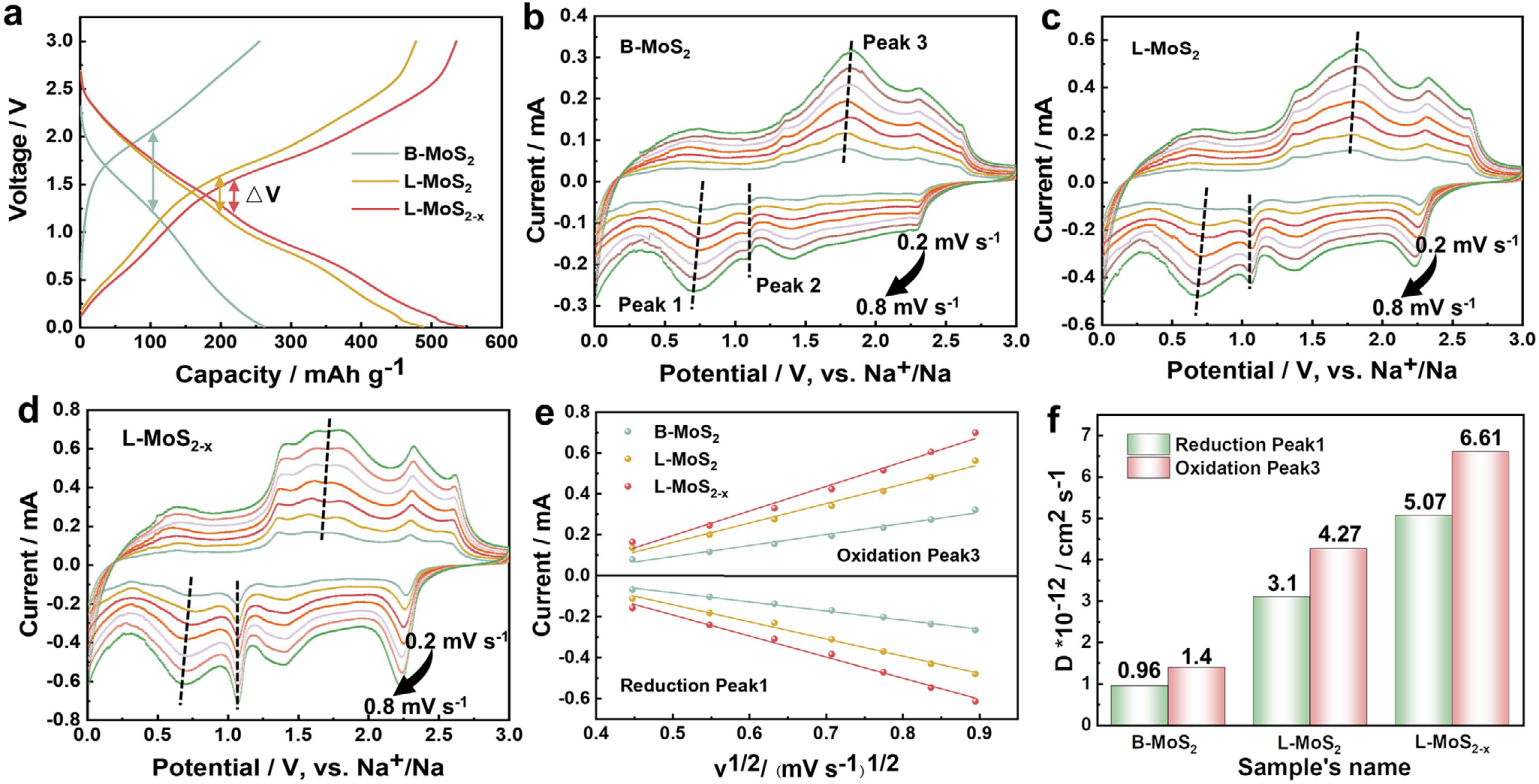
Na^+^ kinetic performance of materials. a) The 30th galvanostatic charge/discharge (GCD) curves of B‐MoS_2_, L‐MoS_2_, and L‐MoS_2‐x_. b–d) CV curves of B‐MoS_2_, L‐MoS_2_, and L‐MoS_2‐x_ at stepwise scan rates from 0.2 to 0.8 mV s^−1^. e) The fitted I_P_/v^1/2^ curves. f) *D*_Na+_ of reduction peak 1 and oxidation peak 3.

The impressive electrochemical performances of L‐MoS_2‐x_ in sodium‐ion batteries prompted a comprehensive evaluation of the detailed electrochemical kinetics for this as‐obtained electrode. The 30th galvanostatic charge/discharge (GCD) curve (**Figure** **3**a) demonstrates the relatively low voltage gaps (ΔV) for L‐MoS_2‐x_, indicating the decreased ohmic/electrochemical polarization due to the promoted electron/ion transfer rate and enhanced electrochemical kinetics. The diffusion coefficient of Na‐ions (*D*_Na+_) in the as‐prepared B‐MoS_2_, L‐MoS_2_, and L‐MoS_2‐x_ electrodes was measured from CV curves (vs Na/Na^+^) in a scan range of 0.2‐0.8 mV s^−1^ (Figure 3b–d). Apparently, the reduction peak 1 and oxidation peak 3 of L‐MoS_2‐x_ exhibit higher values than those of B‐MoS_2_ and L‐MoS_2_ at the same high scanning rate, proving better reaction kinetics of L‐MoS_2‐x_. Additionally, the the diffusion coefficients (*D*_Na+_) are calculated by fitting the slopes of the Ip/v^1/2^ curves corresponding to the two redox peaks, yielding values in the following order: B‐MoS_2_ (0.956×10^−12^/1.4×10^−12^ cm^2^ s^−1^) < L‐ MoS_2_ (3.1×10^−12^/4.27×10^−12^ cm^2^ s^−1^) < L‐MoS_2‐x_ (5.07×10^−12^/6.61×10^−12^ cm^2^ s^−1^), as shown in Figure 3e,f. The relatively high *D*_Na+_ observed for L‐MoS_2‐x_ demonstrates that the enhanced Na–ion transfer rates during redox reactions could be attributed to the structure design of S‐vacancies, thereby demonstrating its ultrafast Na‐ion storage capability. Furthermore, to better confirm the Na^+^ diffusion behaviors, galvanostatic intermittent titration technique (GITT) was performed on the as‐obtained samples between 0.01 and 3 V, and *D*_Na+_ at different discharge/charge potentials were calculated during this process. As displayed in Figure S22 (Supporting Information), the L‐MoS_2‐x_ exhibits the largest *D*_Na+_ across the potential range from 0.01 to 3 V, further verifying that the edge S vacancy design of the MoS_2_ electrode could enhance rapid ion diffusion and transfer, thereby boosting their electrochemical performance in sodium ion storage.

**Figure 4:**
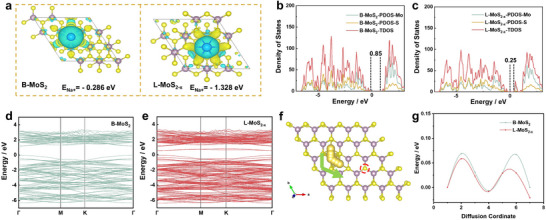
DFT calculations. a) The charge density difference of Na adsorption, and the corresponding adsorption energy on B‐MoS_2_ and L‐MoS_2‐x_. Total density of state: b) B‐MoS_2_ and c) L‐MoS_2‐x_. The band structures of d) B‐MoS_2_ and e) L‐MoS_2‐x_. f)The Na diffusion pathways of L‐MoS_2‐x_. g) The corresponding migration energy of Na on B‐MoS_2_ and L‐MoS_2‐x_.

Density functional theory (DFT) calculations were thus conducted to gain insight into the internal mechanism of L‐MoS_2‐x_ on the high electrochemical performance at the molecular level. As demonstrated from the isosurface of local charge density difference of Na adsorption (**Figure** **4**a; Figure S23, Supporting Information), L‐MoS_2‐x_ exhibits superior electron‐deficient properties in terms of electron density during Na^+^ adsorption with enhanced charge compensation compared to vacancy‐free sites. Furthermore, the adsorption energy of a sodium atom on L‐MoS_2‐x_ is calculated to be −1.328 eV, significantly lower than that on MoS_2_ (−0.286 eV), indicating a higher affinity for sodium anchoring on S‐deficient MoS_2_ compared to normal MoS_2_, which contributes to the enhanced sodium storage capabilities. The electronic structures of L‐MoS_2‐x_ and MoS_2_ are illustrated in Figure 4b,c, respectively. Compared to MoS_2_ (0.85 eV), a reduced bandgap of 0.25 eV is observed for L‐MoS_2‐x_, indicating enhanced conductivity and accelerated charge transfer. In addition, as depicted in Figure 4d,e, the energy band structures among the two samples are well consistent with the TDOS result, displaying a relatively higher electronic conductivity for L‐MoS_2‐x_. The impact of edge S‐vacancies in MoS_2_ on Na^+^ transport kinetics was also investigated by assessing the migration energy associated with the Na^+^ diffusion pathway. Consequently, the lower migration barrier energy for sodium atoms on L‐MoS_2‐x_ is identified by Nudged Elastic Band (NEB) analysis, as shown in Figure 4f,g, indicating the improved reaction kinetics. Besides, the Bader charge calculation was carried out to clarify the local chemical variation while *S* vacancy exists (Figure S24 and Table S1, Supporting Information). Results show that the *S* vacancy will lower the valence state of both Mo and *S*, which is in accordance with the XPS data.

## 3 Conclusion

A facile and low‐energy‐consumption technique of metal ion‐assisted shear exfoliation is developed for the fabrication of 2D MoS_2_ with edge S‐vacancies as an anode material for sodium ion storage. Take advantage of this layer and vacancy structure design, the related issues of low conductivity and capacity, volume variation during cycling, as well as poor reaction kinetics of MoS_2_ have been conquered successfully. The developed L‐MoS_2‐x_ anode demonstrates high electronic conductivity and rapid Na^+^ transport kinetics. The abundant S‐vacancies in L‐MoS_2‐x_ enable the Na‐ion battery to achieve a high specific capacity of 399.91 mAh g^−1^ at a current density of 5 A g^−1^, while exhibiting excellent cycling stability with a capacity retention of 460.71 mAh g^−1^ after 100 cycles at 1 A g^−1^. The utilization of metal ion‐assisted shear‐exfoliation offers a novel pathway for fabricating S‐vacancy‐constructed transition metal disulfides, which possess great potential for application in energy storage.

## Conflict of Interest

The authors declare no conflict of interest.

## Supporting information



Supporting Information

## Data Availability

The data that support the findings of this study are available in the supplementary material of this article.
