# Supplementary material for: Fabricating 2D MoS2 with Edge Sulfur Vacancy Defects by Heavy Ion Bombardment Shear‐Exfoliation for Enhanced Sodium Storage
Source: Adv Sci (Weinh). 2025 Jul 15;12(37):e17576. doi: 10.1002/advs.202417576 (PMC12499421; doi:10.1002/advs.202417576)
Supplement: Supplementary file 1 — Supporting Information [file ADVS-12-e17576-s001.docx]

Supplementary Information

**Fabricating 2D** **MoS_2_ with** **edge sulfur vacancy defects by** **heavy ion bombardment shear-exfoliation for enhanced sodium storage**

Feiyan Mu^[a]^, Siqi Li^[a]^, Dongqi Zhang^[a]^, Qing Zhang^[b]^, Zhe Hu* ^[c]^, Ye Han^[d]^, Zhixin Tai* ^[a]^, Yajie Liu* ^[a]^

[a] F. Mu, S Li, D. Zhang, Z. Tai, Y. Liu

Advanced Energy Storage Materials and Technology Research Center

Guangdong-Hong Kong Joint Laboratory for Carbon Neutrality, Jiangmen Laboratory of Carbon Science and Technology

Jiangmen 529199, China

E-mail: liuyajie@hkustgz-jcl.ac.cn; taizhixin@hkustgz-jcl.ac.cn; huzhe@szu.edu.cn

[b] Q. Zhang

Shenzhen Key Laboratory of Advanced Energy Storage, Department of Mechanical and Energy Engineering

Southern University of Science and Technology

Shenzhen 518055, China

[c] Z. Hu

Guangdong Provincial Key Laboratory of Service Safety for New Energy Materials

College of Materials Science and Engineering, Shenzhen University

Shenzhen 518055, China

[d] Y. Han

School of Science

Changchun University

Changchun 130022, China

# These authors contributed equally to this work.

Supporting information for this article is given via a link at the end of the document.

**Experiment**

**Preparation of L-MoS_2_ and L-MoS_2-x_.**

The B-MoS_2_ and Co (NO_3_)_2_ were purchased from Aladdin Co. Ltd., and while anhydrous ethanol was obtained from Inocet Co. Ltd.. The L5M high-shear laboratory mixer utilized in this study is made by Silverson Machines Ltd., UK. The B-MoS_2_ (1g) were dispersed in a solvent mixture of water and ethanol (V water: V ethanol = 4:3, total volume 400 mL), followed by vigorous stirring for 2 hours to ensure mixed well. Subsequently, the mixer head was carefully lowered and immersed into the solution (placed under ice bath), and then rotated at 6000 rpm for 1 hour, 2 hours, 3 hours respectively. Following each one-hour exfoliation, a resting interval of 30 minutes was conducted. The solutions obtained at different times of exfoliation were allowed to stand at room temperature for 24 hours, after which, the solutions were pumped and filtered. The as-prepared powders were obtained after drying at 100 ℃ for 12 hours in a vacuum drying oven, labled as L-MoS_2_-1, L-MoS_2_-2, L-MoS_2_-3. The sample of L-MoS_2_-2 also can be abbreviated as L-MoS_2_ in the manuscript.

Since the direct addition of salt additive in solution for exfoliation could cause potential aggregation and sedimentation issues during the subsequent resting process (Figure S25a), we employed a different approach to fabricate L-MoS_2_-x. Specifically, Co(NO_3_)_2_ was added into the layer-structured L-MoS_2_ dispersion obtained from the upper liquid solution after the initial exfoliation mentioned above (Figure S25b). Here, the solutions obtained at different times of first-step exfoliation were allowed to stand at room temperature for 24 hours, and then the supernatant liquid (200mL) was taken and added with different masses of Co(NO_3_)_2_ (2g, 6g, 10g, 14g), and followed by the second-step exfoliation for 30 minutes. Afterward, the obtained dispersions were filtered and washed. and the resulting powder can be obtained after dried at 100 ℃ for 12 hours in vacuum drying oven. This powder can be labled as L-MoS_2-x_-1, L-MoS_2-x_-2, L-MoS_2-x_-3, and L-MoS_2-x_-4. In manuscript, the sample of L-MoS_2-x_-3 can also be abbreviated as L-MoS_2-x_ for comparation with other counterparts.

**Characterization of materials.**

The microstructures of sample were examined using field emission scanning electron microscopy (FE-SEM, ZEISS Sigma 300) and high-resolution TEM (HRTEM, FEI Talos F200x). The presence of S vacancies was visualized by spherical aberration electron microscopy (STEM, Titan Themis G2) and demonstrated by electron paramagnetic resonance (EPR, Bruker EMXplus-6/1). The elemental composition of the samples was determined though energy dispersive spectroscopy (EDS) and X-ray photoelectron spectroscopy (XPS, Thermo Scientific K-Alpha). The chemical structure of the samples was obtained via X-ray diffraction (XRD, Rigaku Ultima IV) and Raman spectra (Raman, WITec alpha300R).

**Electrochemical measurements.**

Coin-type (CR2032) cells were equipped with a diaphragm (Whatman, GF/F) and 200 μL of electrolyte, unless otherwise noted. Prior to assembly, circular sheets with 13 mm in diameter were obtained by sectioning the Na metal block (Aladdin Chemical Company). The cathode slurry consisted of MoS_2_, super P and CMC in a mass ratio of 7:2:1. The electrodes were cast onto copper foils and subsequently dried in a vacuum oven at 100 °C for 12 hours. The electrolyte used in this study consisted of 1 M NaClO_4_ dissolved in a solvent mixture comprising ethylene carbonate (EC) and propylene carbonate (PC) (1:1 by volume) with 5.0% fluorinated ethylene carbonate (FEC). In addition, the full cells of sodium ion storage were assembled with L-MoS_2-x_ as anode and layered oxide NFM111 as cathode. Electrochemical measurements were conducted on a Neware battery cycler test system. Galvanostatic charge/discharge was carried out on a Neware battery tester within a voltage range of 0.01–3.0 V. The CV measurements were conducted at a scan rate of 0.1 mV s^−1^ and the EIS measurements were carried out within the frequency range from 100 kHz to 0.01 Hz on a Chenhua CHI660D electrochemical workstation.

**DFT calculations**

All calculations were performed with the Vienna ab-initio Simulation Package (VASP) based on the density functional theory (DFT).^[1]^ The projector-augmented wave (PAW) was used for the electron-ion interactions.^[2]^ The exchange-correlation energy was evaluated using the generalized gradient approximation (GGA) functional of Perdew, Burke, and Enzerhof (PBE) was employed to evaluate the exchange-correlation energy.^[3]^ The semi-empirical London dispersion corrections of Grimme et al. were used to calculate the interactions between absorbers and samples.^[4]^ The cut-off energy of 520 eV was set with a 4 x 4 x 1 Gamma centered k-point grid. The energy convergence tolerance was set below 1 × 10^-5^ eV/atom. The lattice vectors and atomic coordinates were relaxed until the Hellmann-Feynman force on each atom was reduced to less than 0.02 eV/Å.


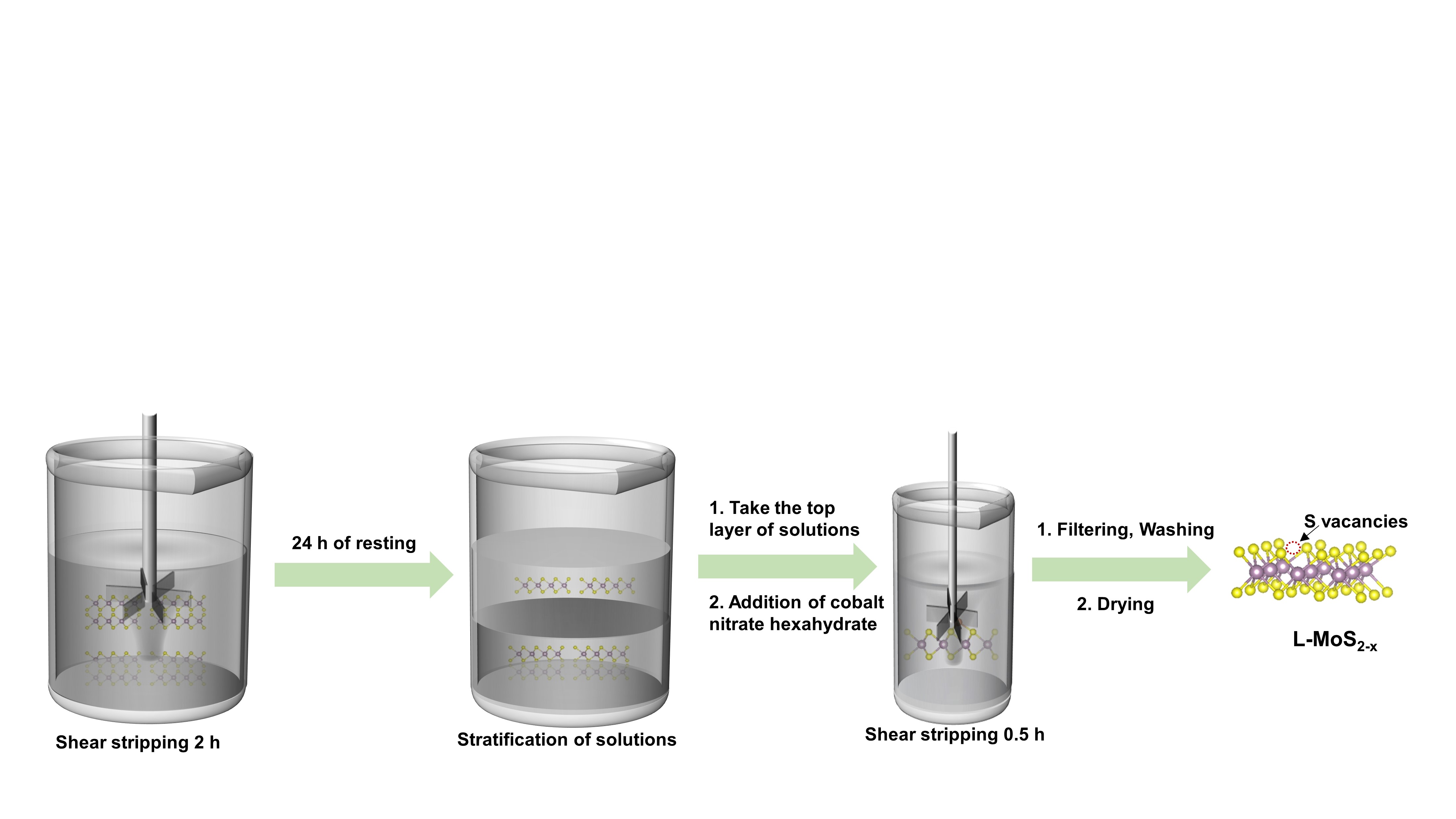


**Figure S1**. Flow chart for synthesizing L-MoS_2-x_.


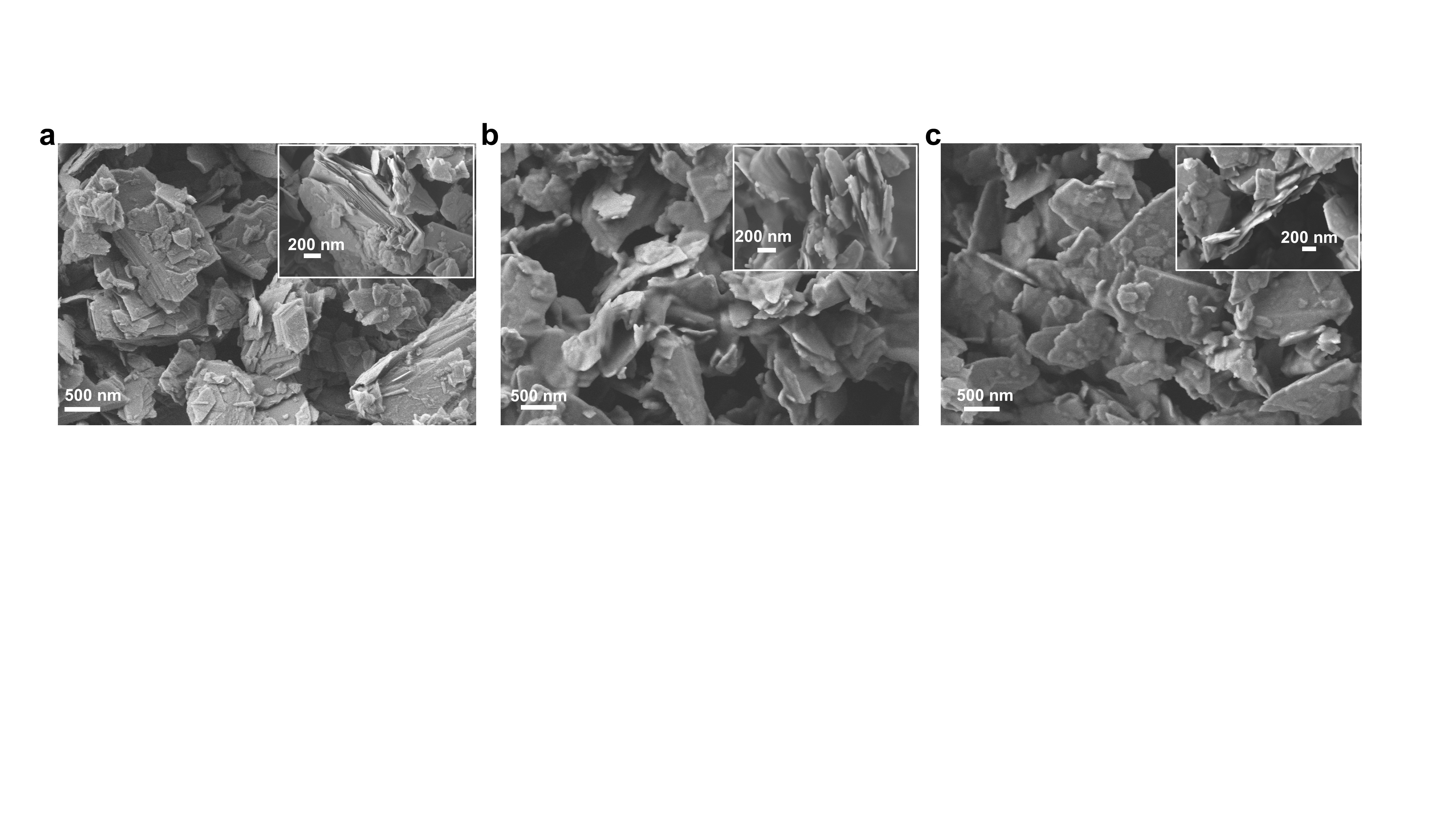


**Figure S2**. SEM characterization (a) B-MoS_2_, (b) L-MoS_2_ (c) L-MoS_2-x_


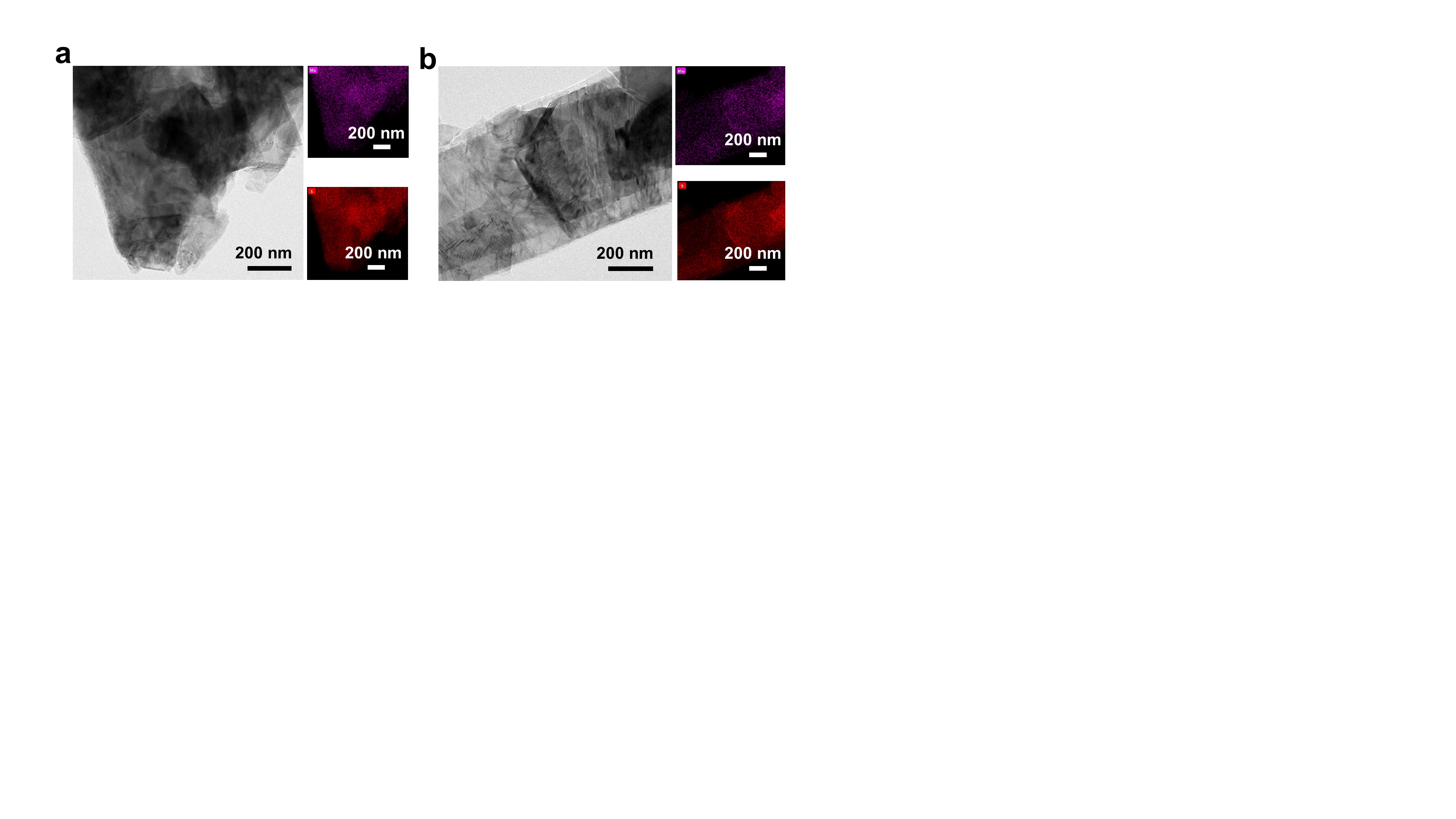


**Figure S3**. TEM characterization of (a) B-MoS_2_, (b) L-MoS_2_.


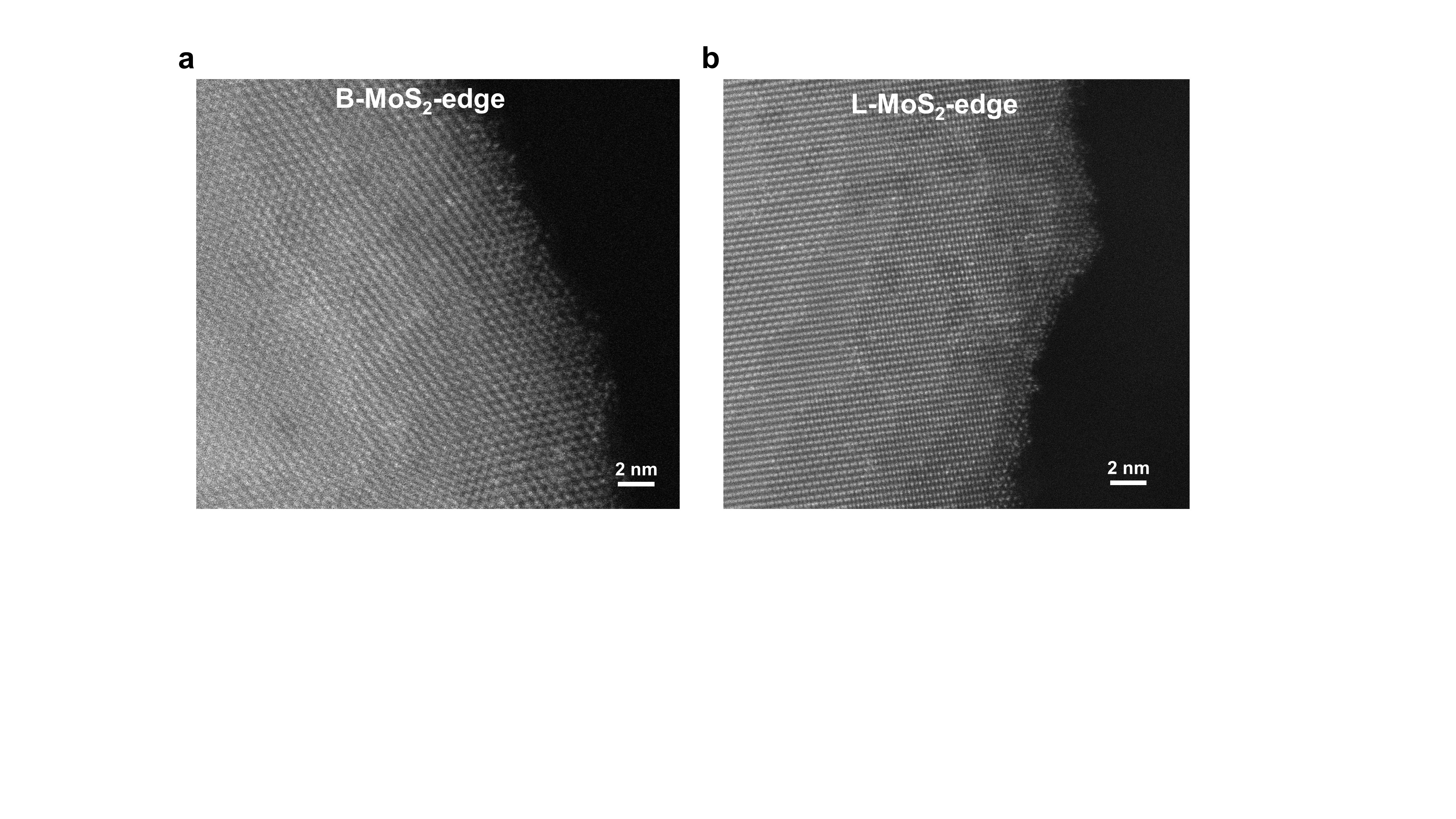


**Figure S4**. Spherical aberration-corrected scanning transmission electron microscopy (STEM) of the B-MoS_2_ and L-MoS_2_.


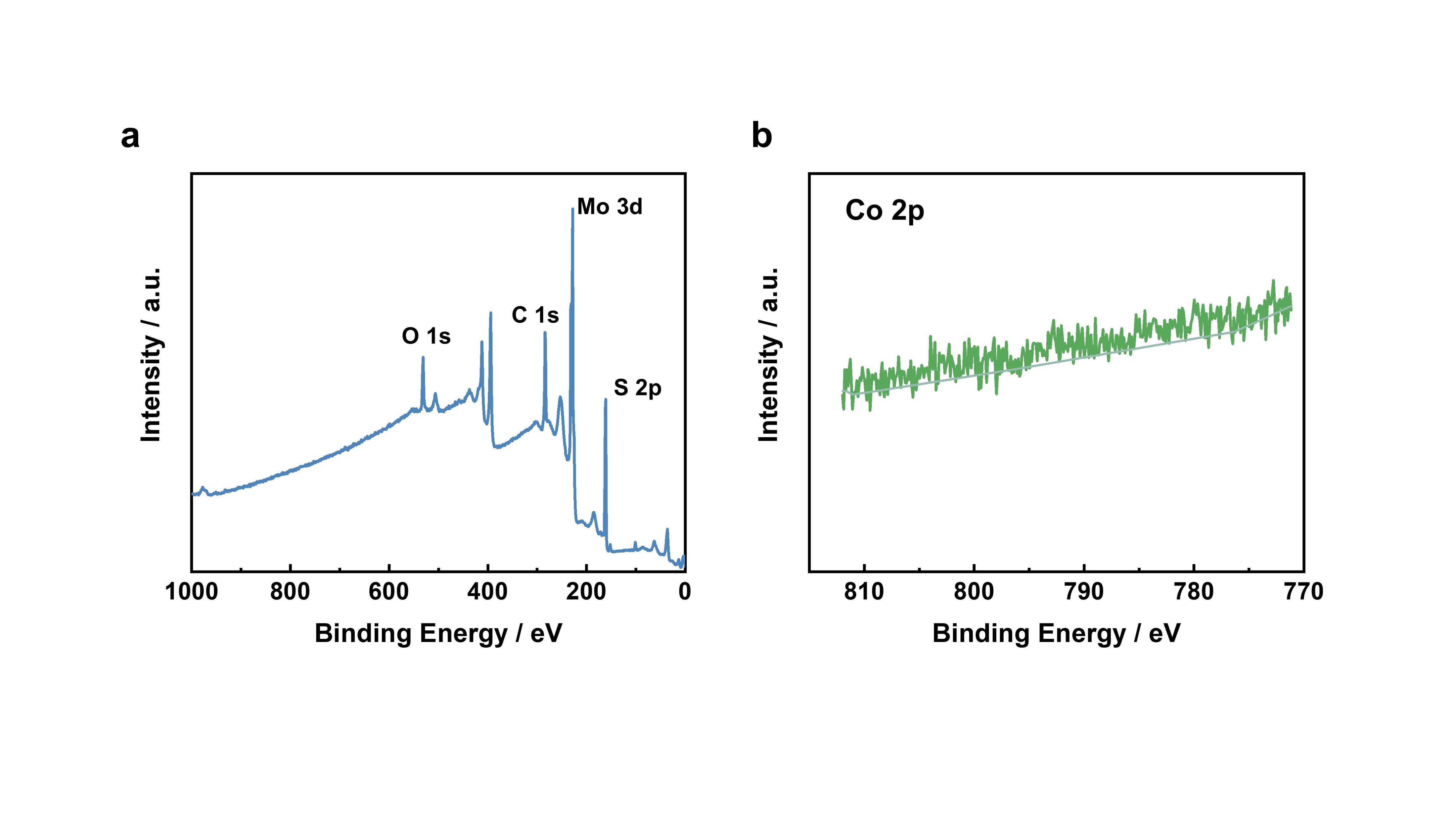


**Figure S5.** (a) High-resolution XPS spectrum of L-MoS_2-x_; (b) XPS of Co 2p spectra for L-MoS_2-x_. The XPS clearly demonstrate the absence of any discernible peaks that could be attributed to Co metal dopants,


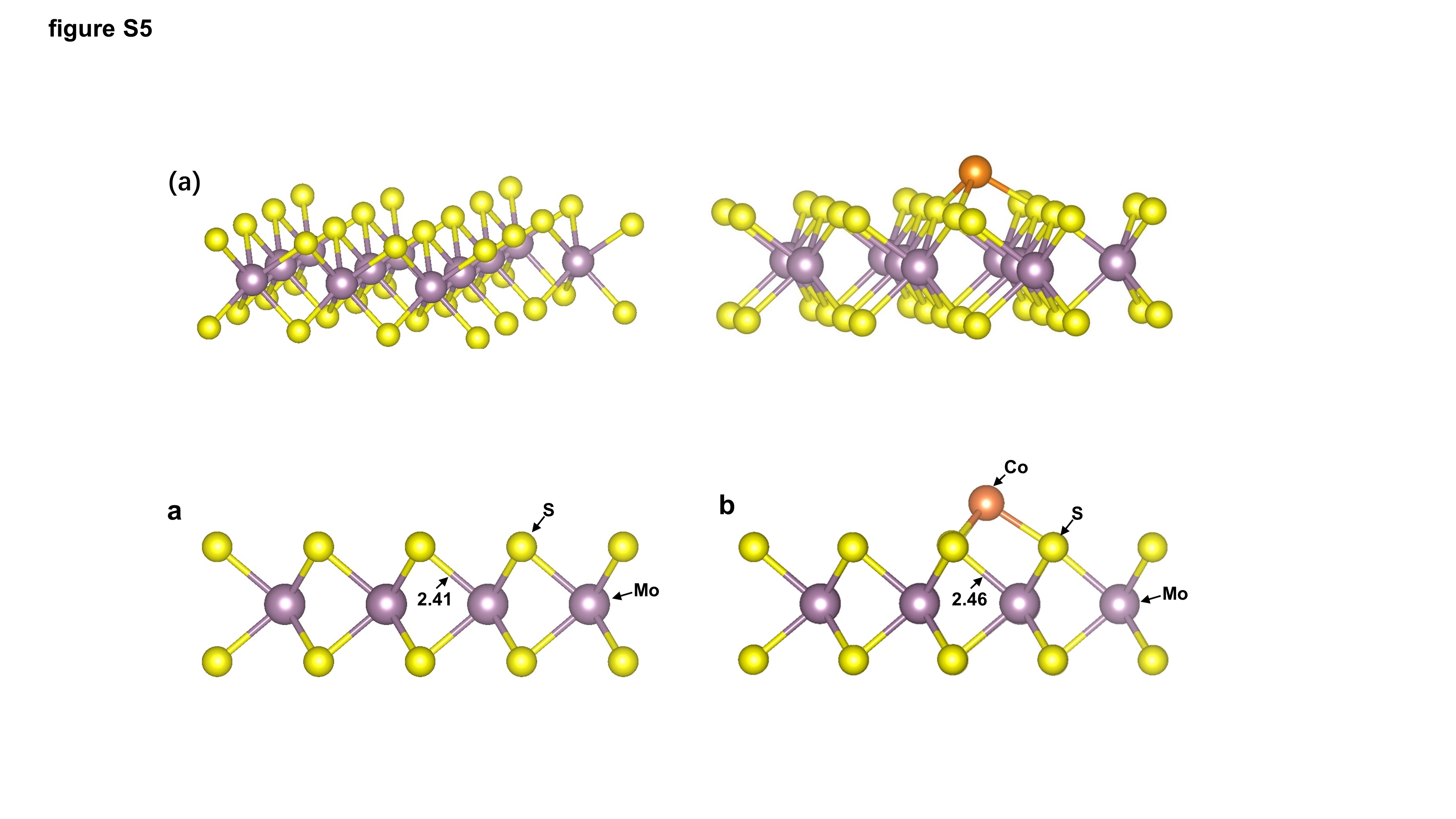


**Figure S6**. Bond length of samples: (a) MoS_2_: 2.41Å b) L-MoS_2_-Co^2+^: 2.46 Å.


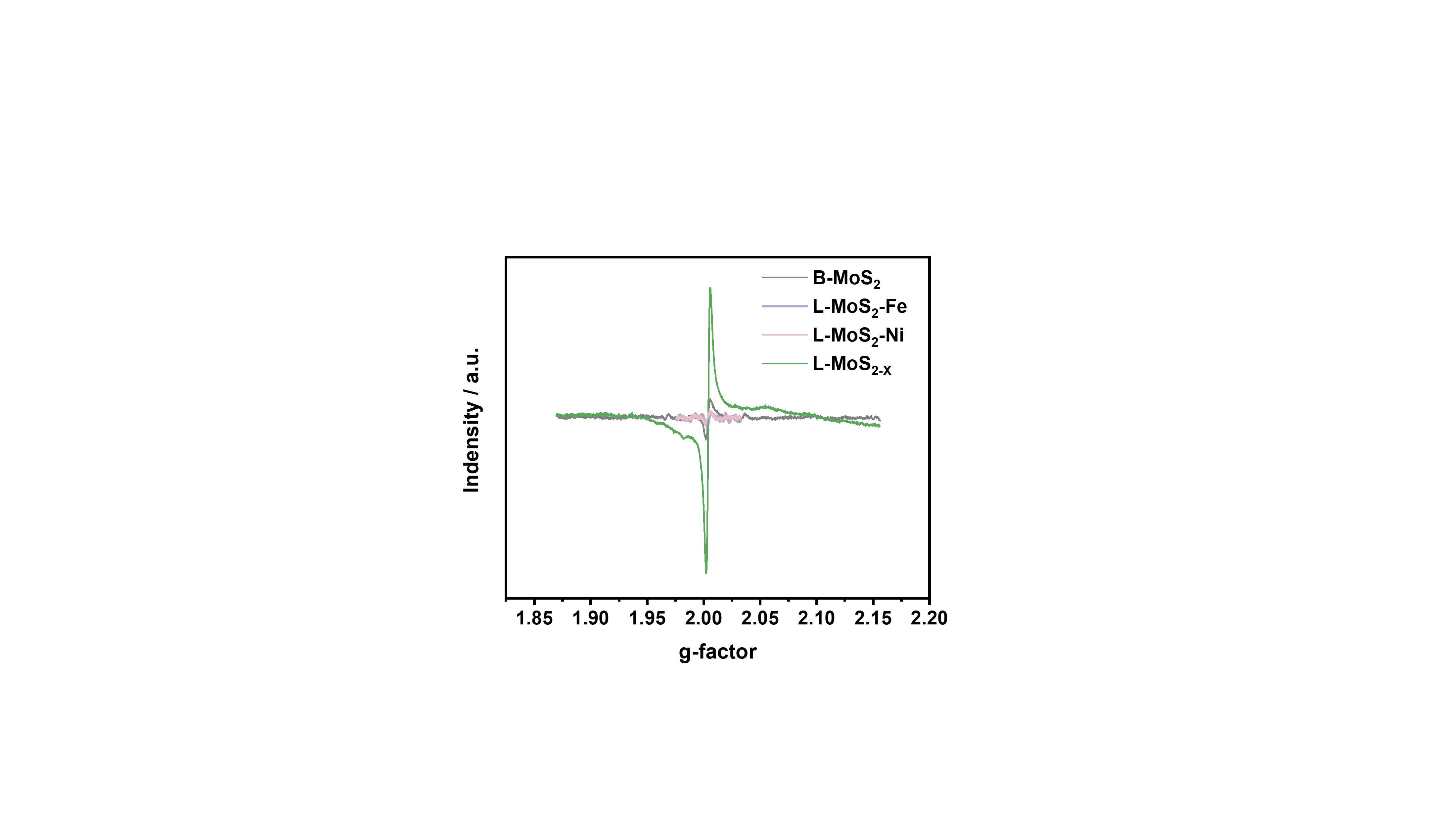


**Figure S7.** Electron paramagnetic resonance (EPR) characterizations of B-MoS_2_, L-MoS_2_-Fe, L-MoS_2_-Ni and L-MoS_2-x_


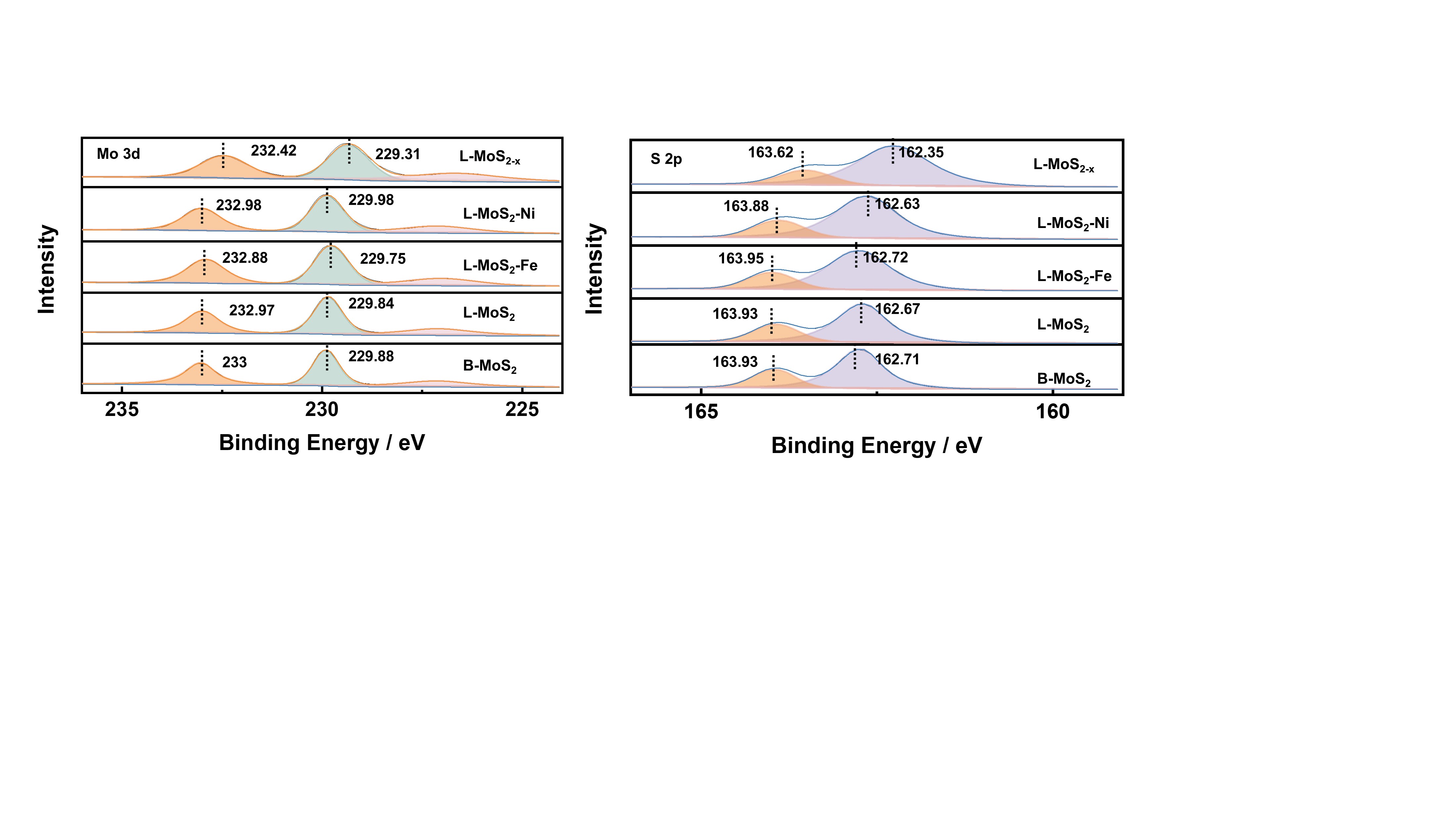


**Figure S8.** High-resolution Mo 3d and S 2p spectrum of the resultant MoS_2_ nanosheets using Ni(NO₃)₂ and Fe(NO₃)₃ as solvent additives.


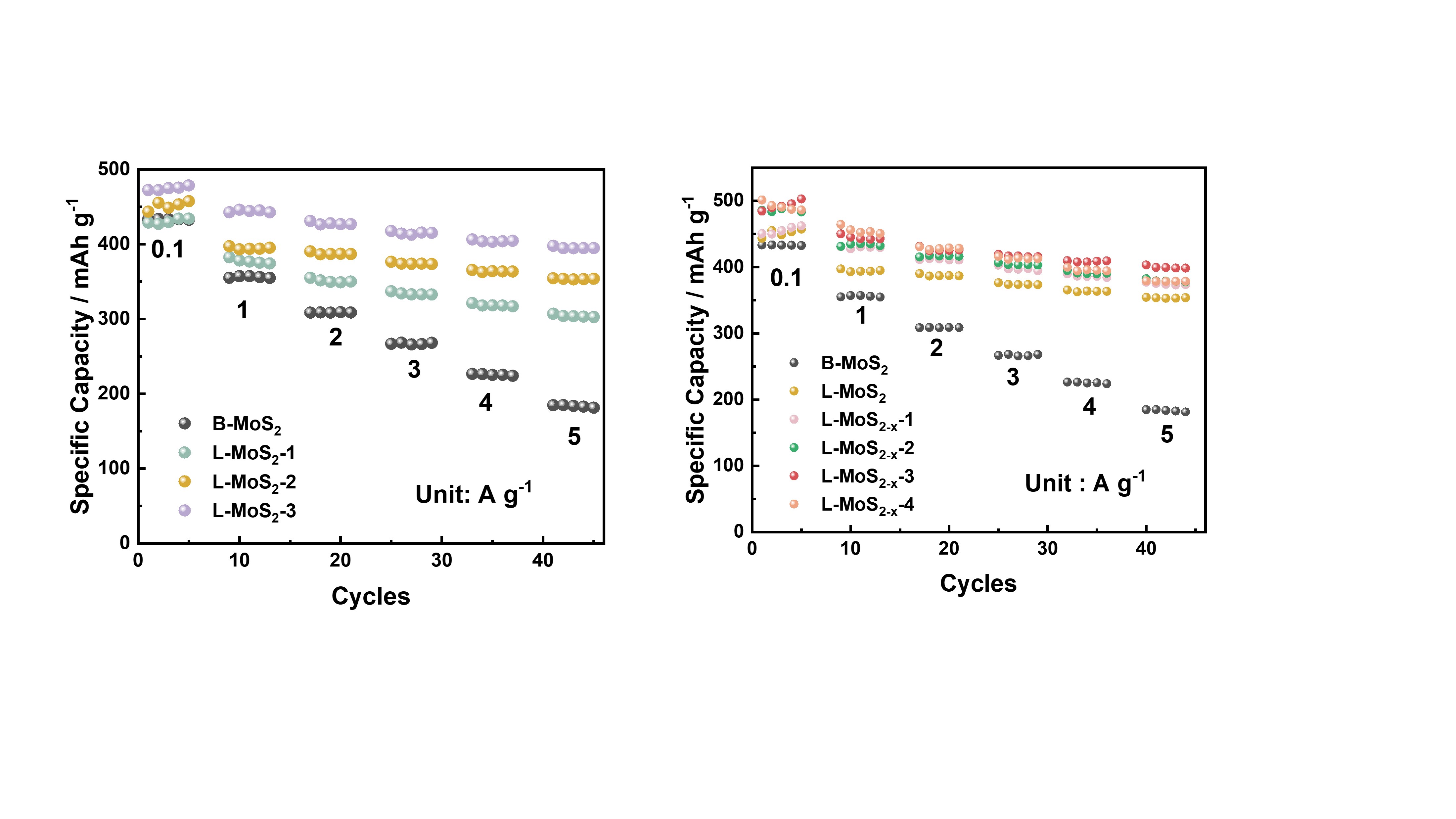


**Figure S9.** The rate performance of B-MoS_2_, L-MoS_2_-1, L-MoS_2_-2, and L-MoS_2_-3 (L-MoS_2_-1, L-MoS_2_-2, and L-MoS_2_-3 indicate 1 hour, 2 hour and 3 hour of exfoliation time, respectively).


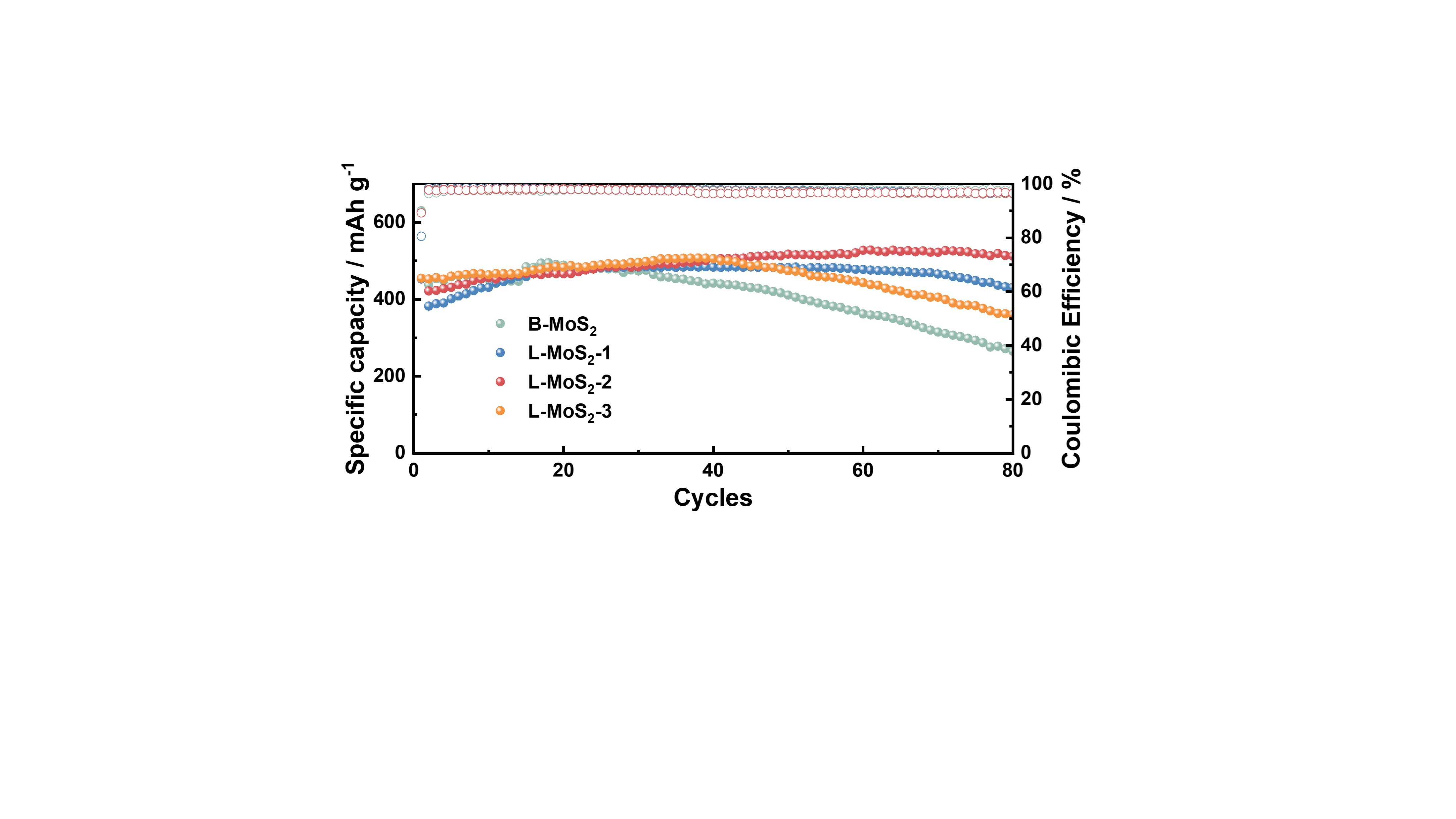


**Figure S10.** The cycling performance of B-MoS_2_, L-MoS_2_-1, L-MoS_2_-2, and L-MoS_2_-3 at the current density of 0.1 A g^-1^.


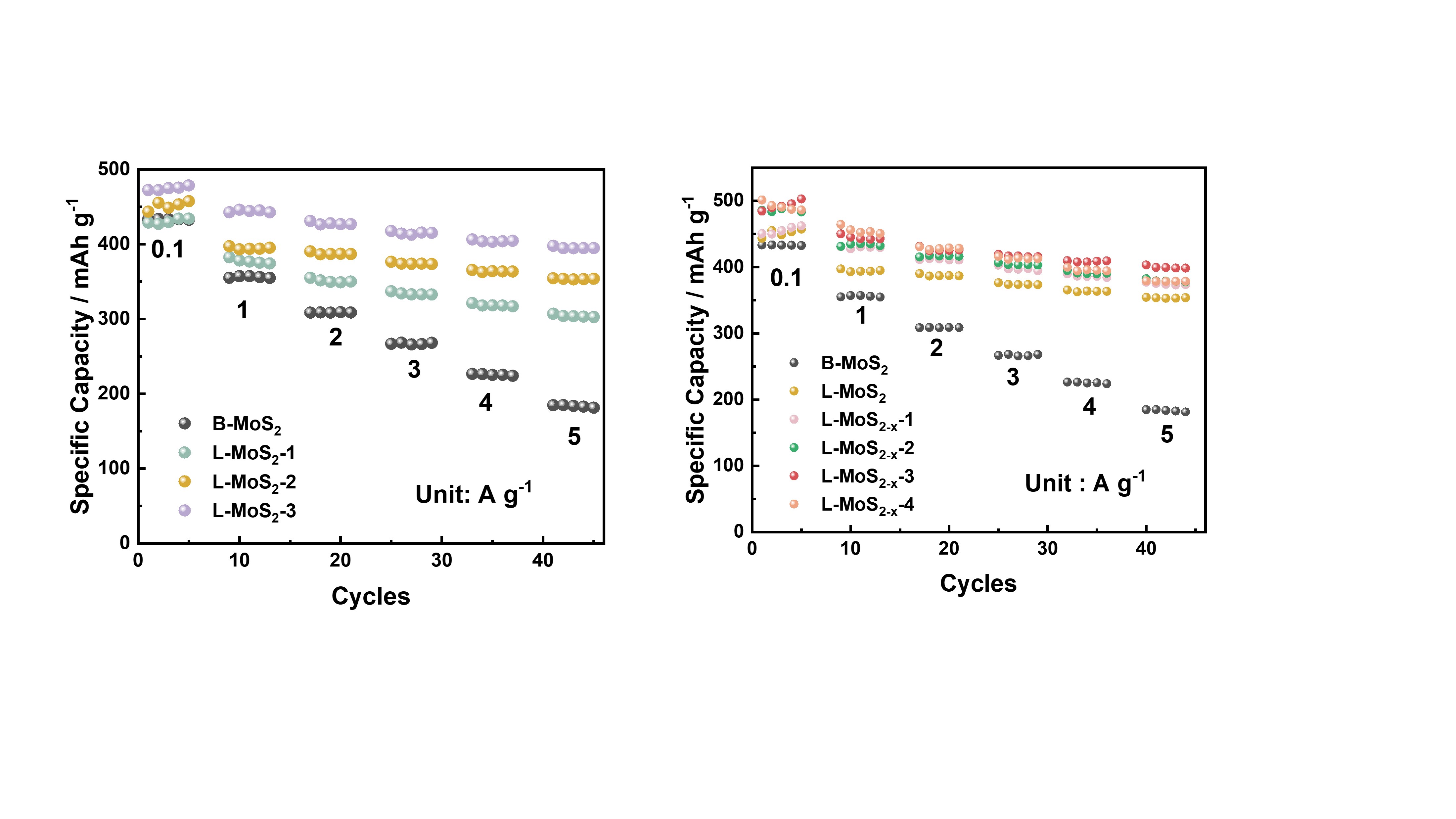


**Figure S11.** The rate performance of B-MoS_2_, L-MoS_2_, L-MoS_2-x_-1, L-MoS_2-x_-2, L-MoS_2-x_-3, and L-MoS_2-x_-4 (L-MoS_2-x_-1, L-MoS_2-x_-2, L-MoS_2-x_-3, L-MoS_2-x_-4 represent the concentrations of Co(NO_3_)_2_ solution : 10 g/L, 30 g/L, 50 g/L, and 70 g/L , respectively.


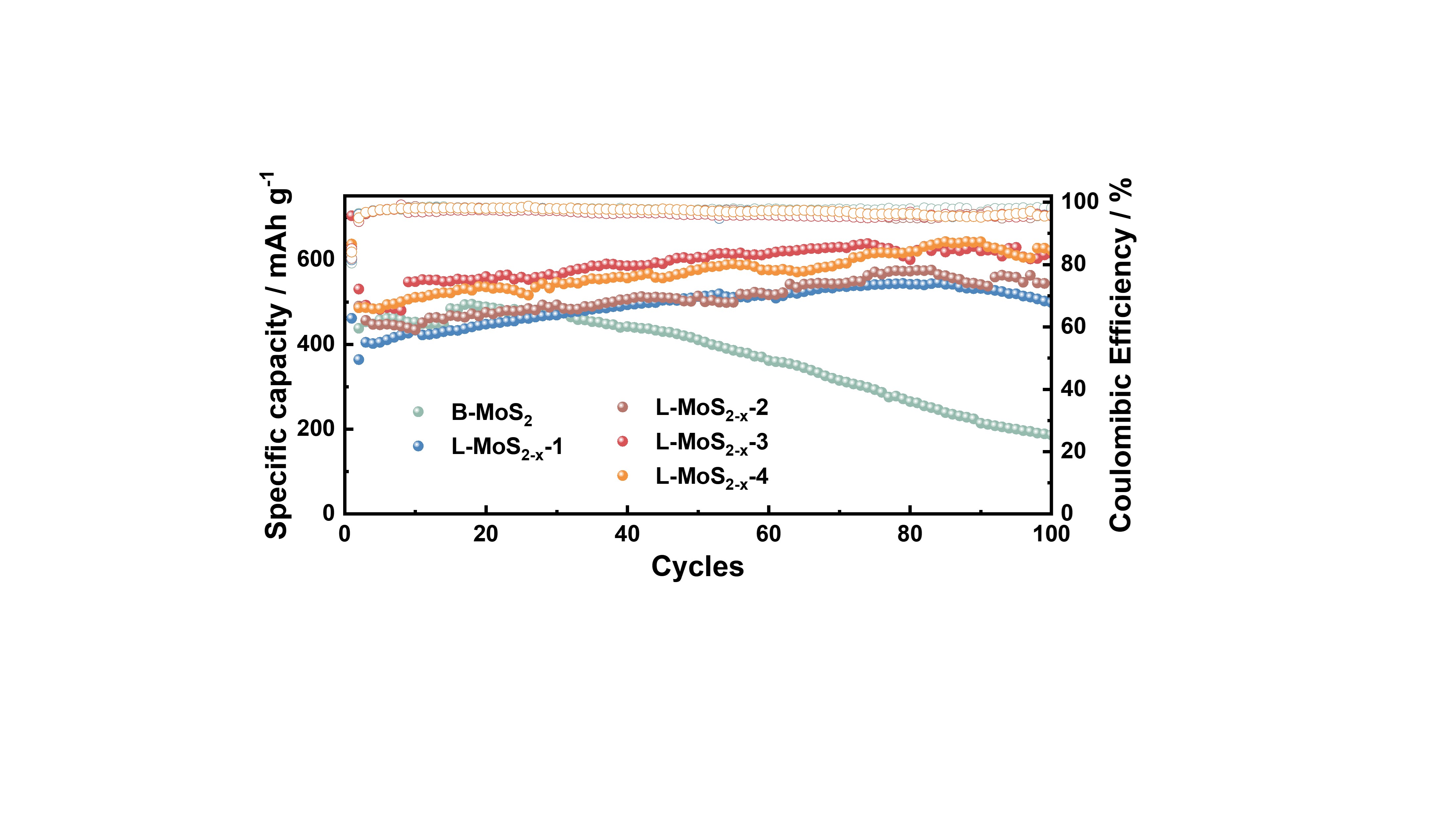


**Figure S12**. The cycling performance of B-MoS_2_, L-MoS_2_, L-MoS_2-x_-1, L-MoS_2-x_-2, L-MoS_2-x_-3, and L-MoS_2-x_-4 at the current density of 0.1 A g^-1^.


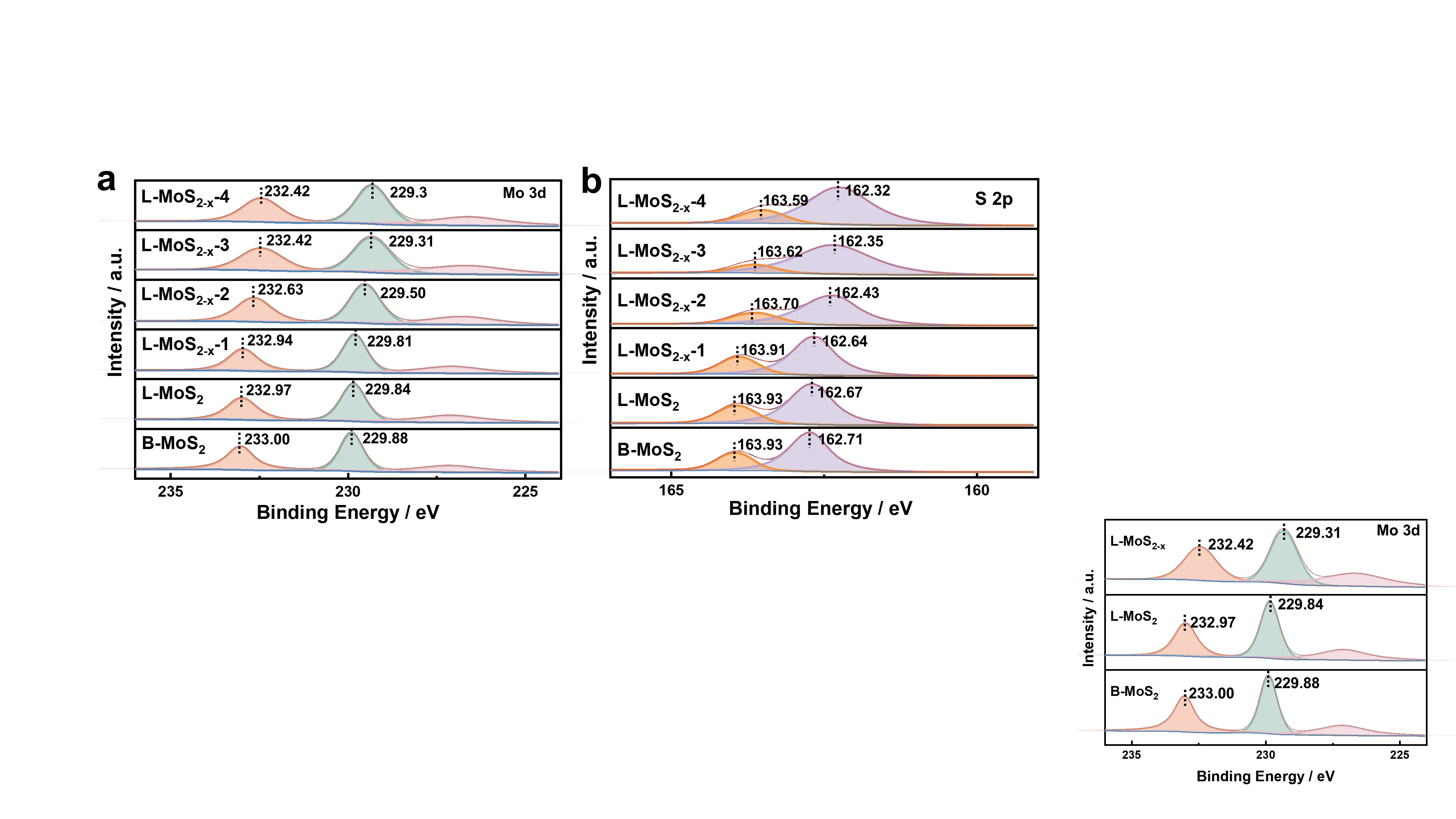


**Figure S13.** (a) High-resolution Mo 3d spectrum of MoS_2_, L-MoS_2_, L-MoS_2-x_-1, L-MoS_2-x_-2, L-MoS_2-x_-3 and L-MoS_2-x_-4. (b) High-resolution S 2p spectrum of of MoS_2_, L-MoS_2_, L-MoS_2-x_-1, L-MoS_2-x_-2, L-MoS_2-x_-3 and L-MoS_2-x_-4, respectively. From the XPS spectra, it is showed that the main peaks of L-MoS_2-x_-2, L-MoS_2-x_-3 gradually shift to lower binding energies compared to L-MoS_2-x_-1, indicating a gradual increase in the number of S vacancies during the assisted shear exfoliation Conversely, there is almost no peak shifts for the L-MoS_2-x_-4 when compared to L-MoS_2-x_-3, indicating a optimal concentration of Co (NO_3_)_2_ in the solvent was determined to be 50 g/L.


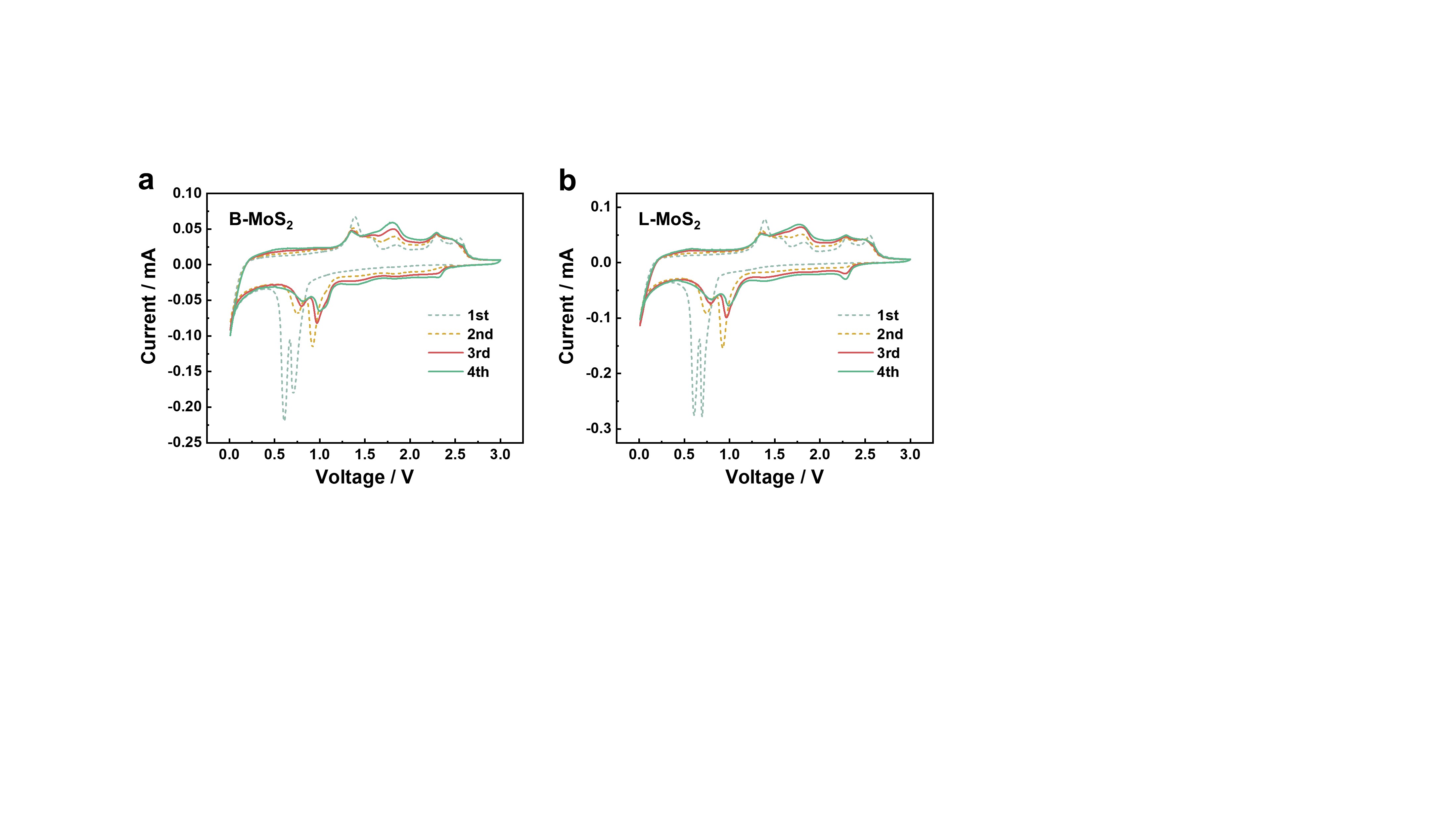


**Figure S14.** CV curves at 0.1 mV s^-1^: (a) B-MoS_2_ (b) L-MoS_2_.


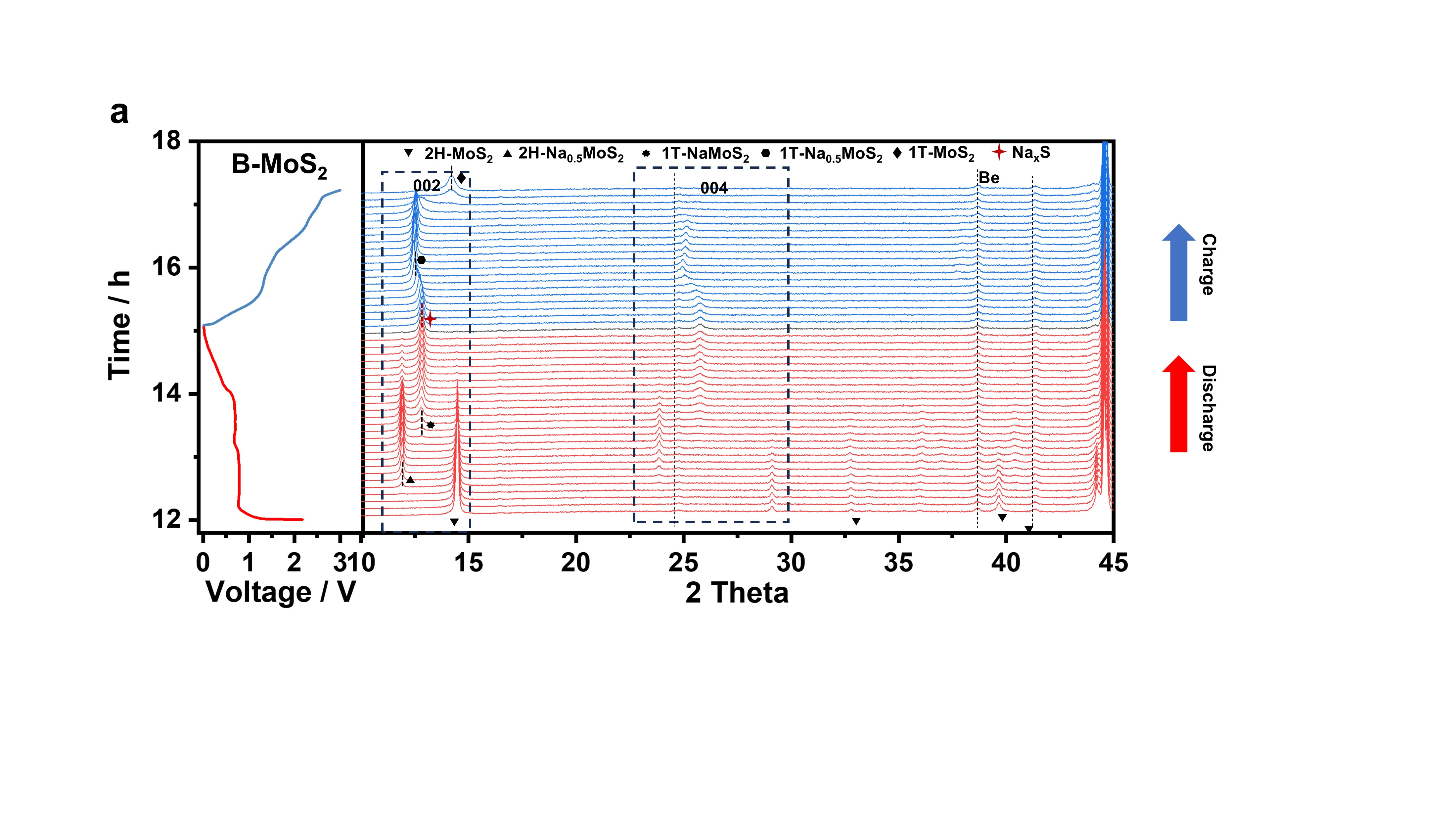

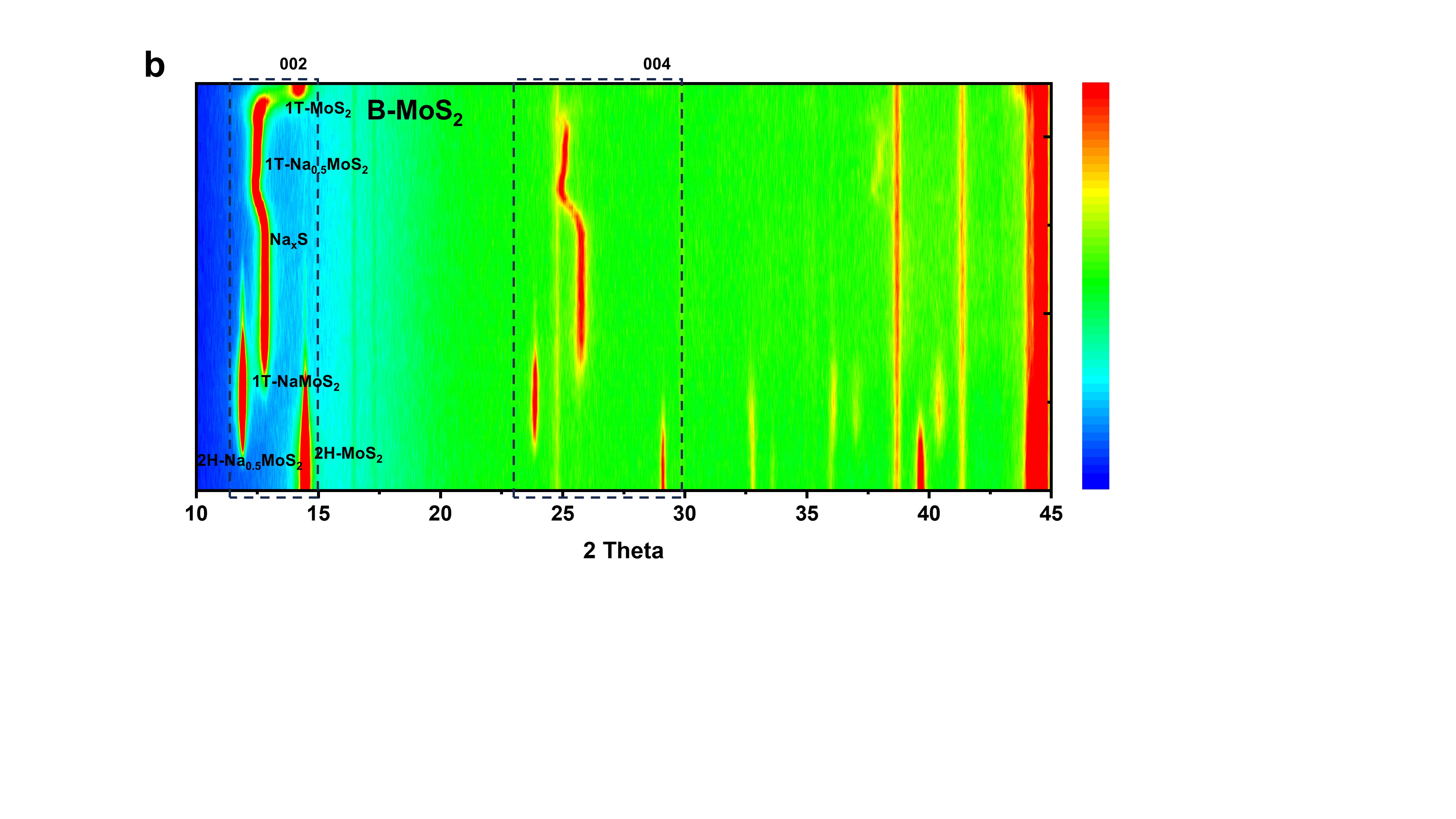

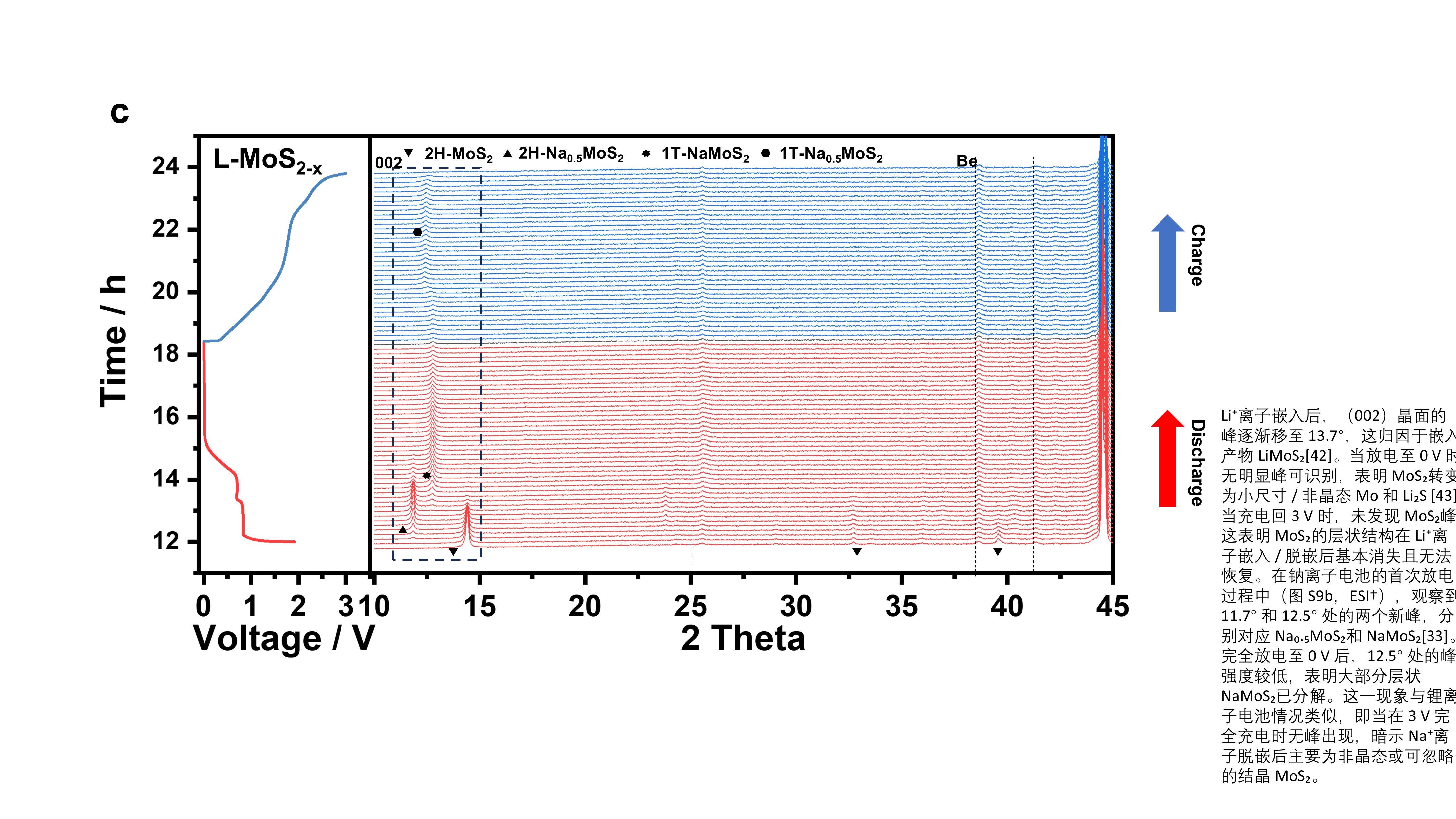

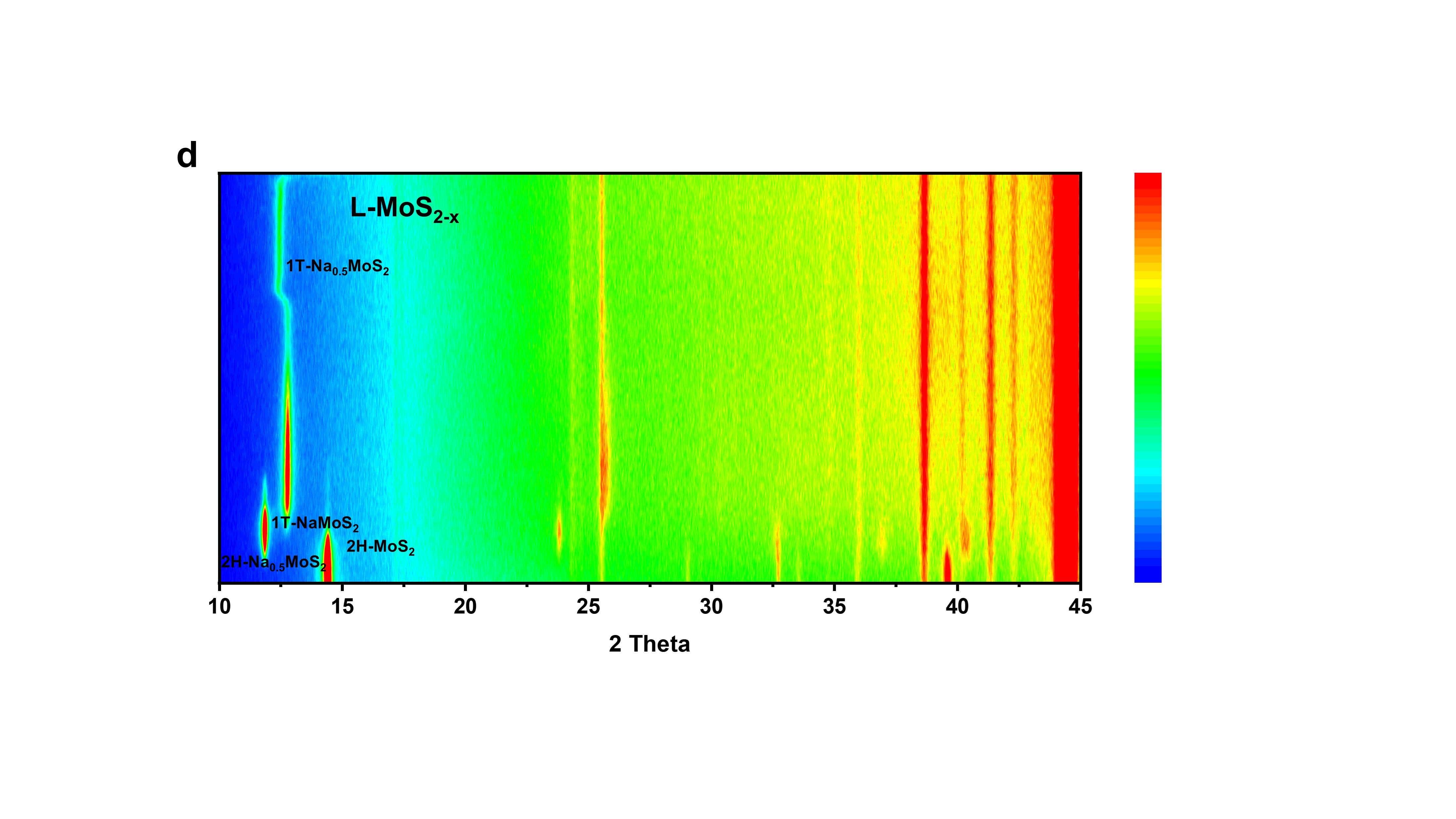


**Figure S15.** In-situ XRD patterns of B-MoS₂ (a,b) and L-MoS₂₋ₓ (c,d) during the first sodiation and desodiation process.

In general, for both of the microsized B-MoS_2_ and L-MoS_2-x_, the reaction mechanism of MoS₂ in sodium-ion batteries can be simply indentified as follows:

MoS₂ + Na⁺ → NaₓMoS₂ (1)

NaₓMoS₂ + 3Na⁺ → Mo + 2Na₂S (2)

Specially, from in situ XRD patterns, it clearly shows that the structural changes of B-MoS₂ and L-MoS₂₋ₓ below 1.00 V involve a two-phase transformation: (where H and T denote hexagonal and tetragonal systems in which MoS_2_ crystallizes)

- Upper plateau (0.85 V): Transition from 2H-MoS₂ (2θ(002) = 14.48°) to 2H-Na₀.₅MoS₂ (2θ(002) = 11.82°);
- Middle plateau (0.75 V): Transition from 2H-Na₀.₅MoS₂ to 1T-NaMoS₂ (2θ(002) = 12.42°).

At the end of discharge, as for B-MoS_2_ electrode, the high-intensity peaks of NaMoS₂ are maintained and the weak characteristic peaks of NaₓS (approximately 2θ = 12.8° and 25.6°) are observed, suggesting the parcially phase transition from 1T-NaMoS₂ to NaxS and nanoscale dispersion of Mo (cannot detected in XRD).^[5]^ While, as for L-MoS_2-x_, after full discharge to 0 V, most of the layered NaMoS_2_ is decomposed, as suggested by the low intensity of the peak at 12.42° and no obvious peaks can be recognized for NaxS, indicating that the L-MoS_2-x_ is almost completely transformed into small/amorphous Mo and NaxS.^[6]^

During the subsequent deintercalation process, the prominent diffraction peaks, the (002) diffraction, for example, shift toward the lower 2θ angles until 1.50 V before going back to higher 2θ angles continuously; no new diffractions are observed in this process. Close to the end of deintercalation (above 2.50 V), a two-phase transition occurs from 1T-Na_0.5_MoS_2_ to 1T-MoS_2_ (2θ(002) = 14.17°) for B-MoS₂ electrode rather than the starting 2H-MoS_2_ (2θ(002) = 14.48°). In compare to that, no peaks can be found when fully charged at 3 V for L-MoS_2-x_, implying that amorphous or negligible crystallized MoS_2_ dominats after Na ion extraction.^[7]^

**
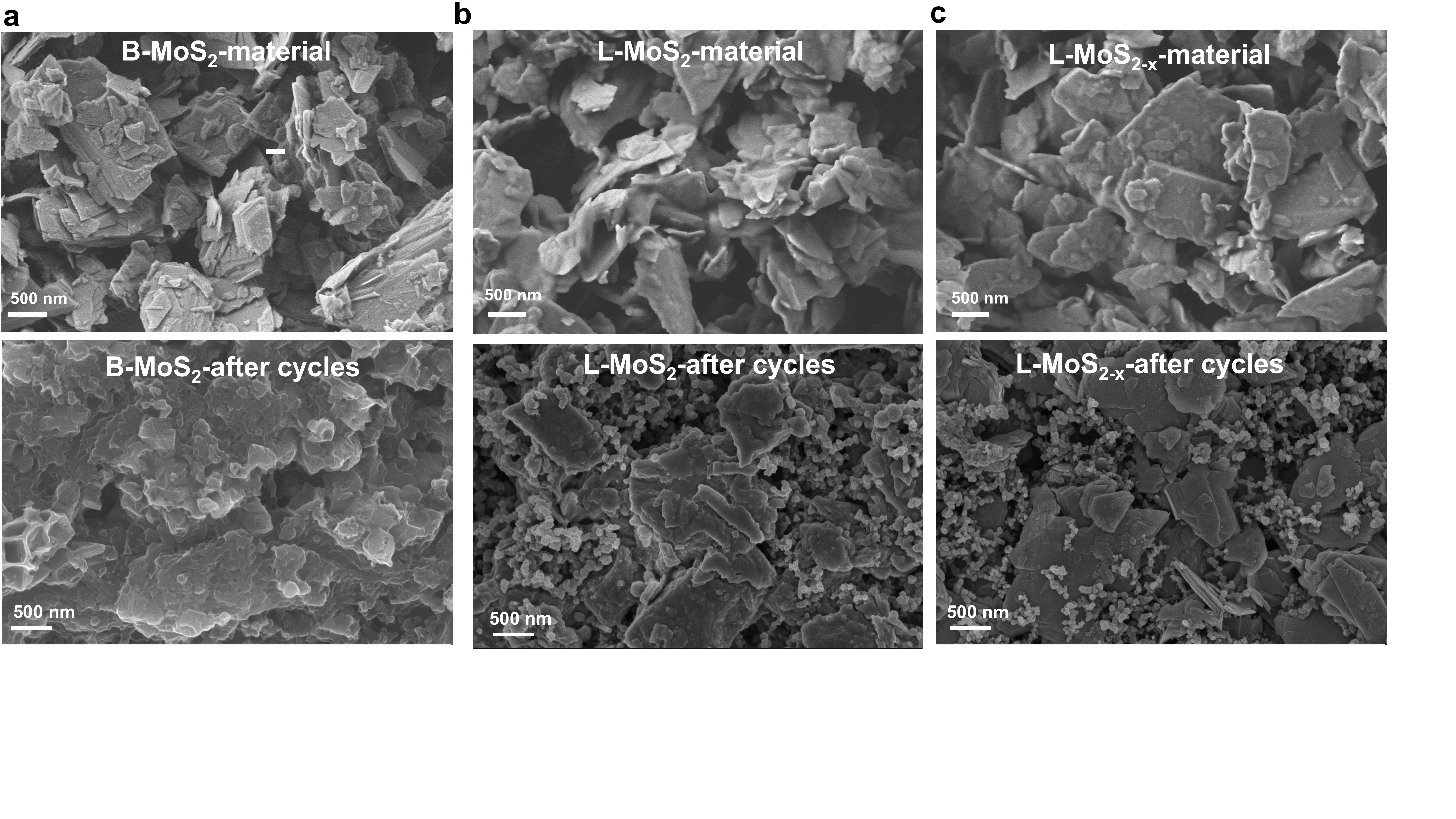
**

**Figure S16.** Surface morphologies of three electrodes (B-MoS_2_, L-MoS_2_ and L-MoS_2-x_) before and after rate tests in Figure 2c.


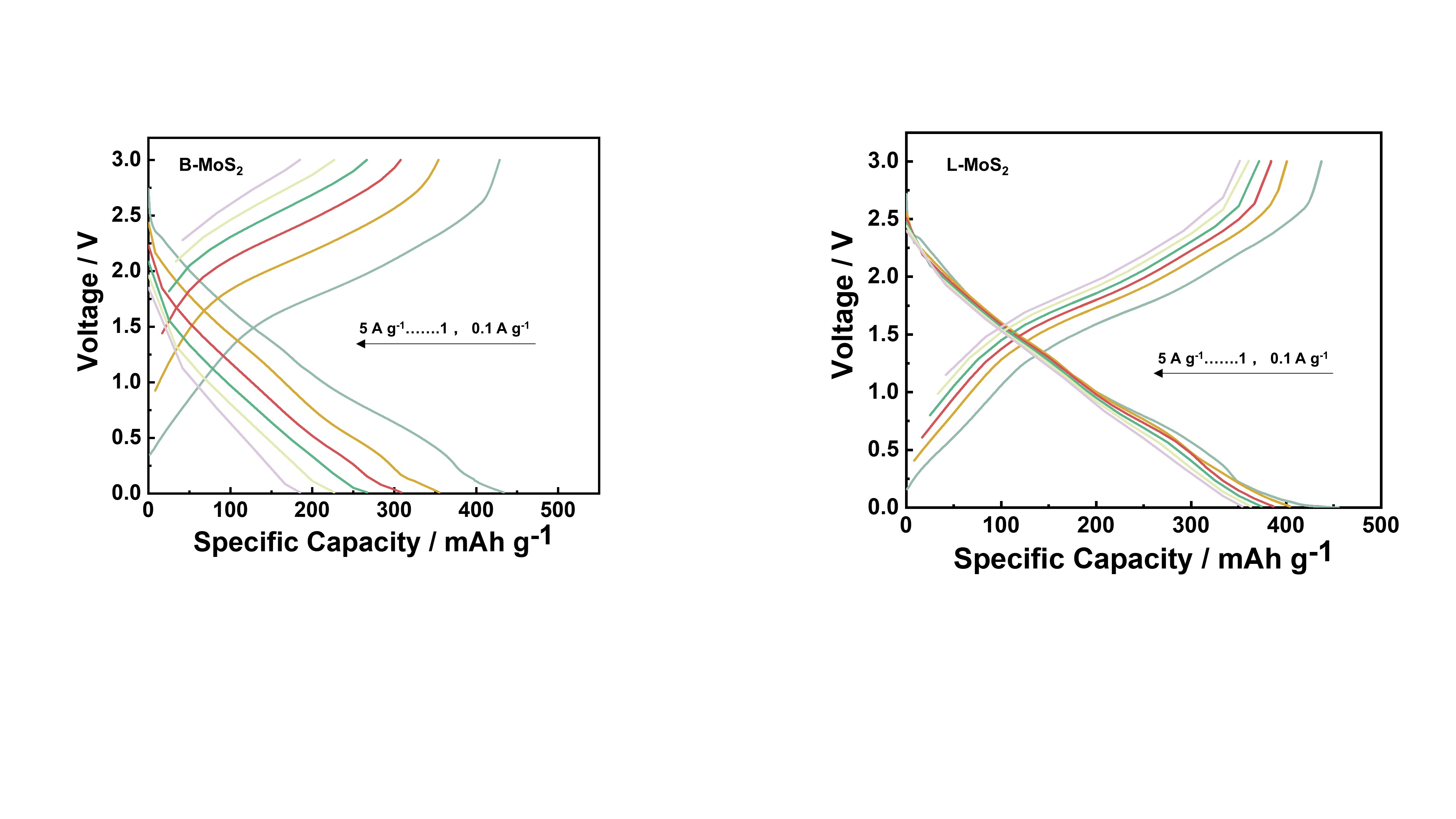


**Figure S17.** Charge-discharge profiles of B-MoS_2_ at diffident rates.


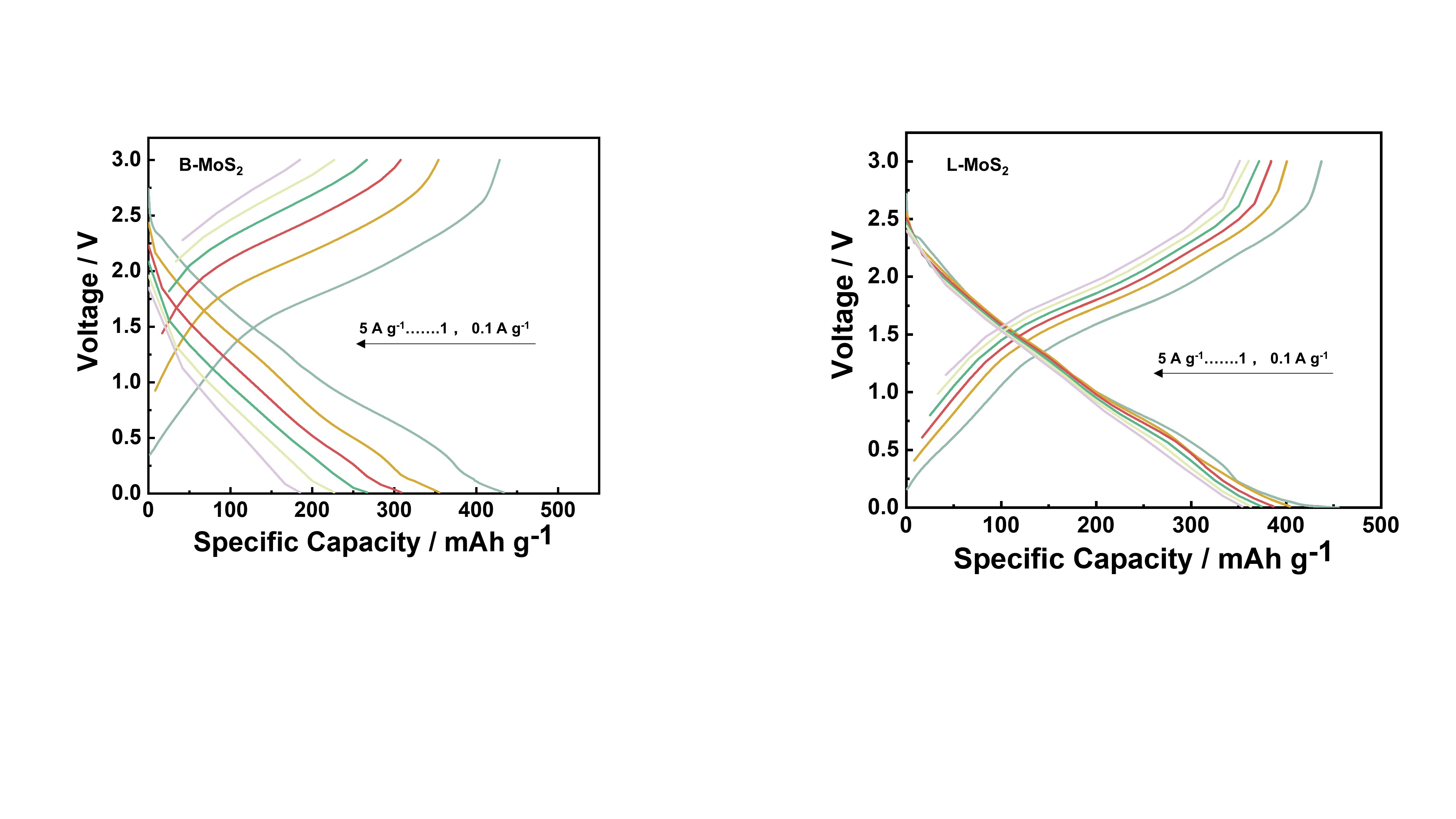


**Figure S18.** Charge-discharge profiles of L-MoS_2_ at diffident rates.


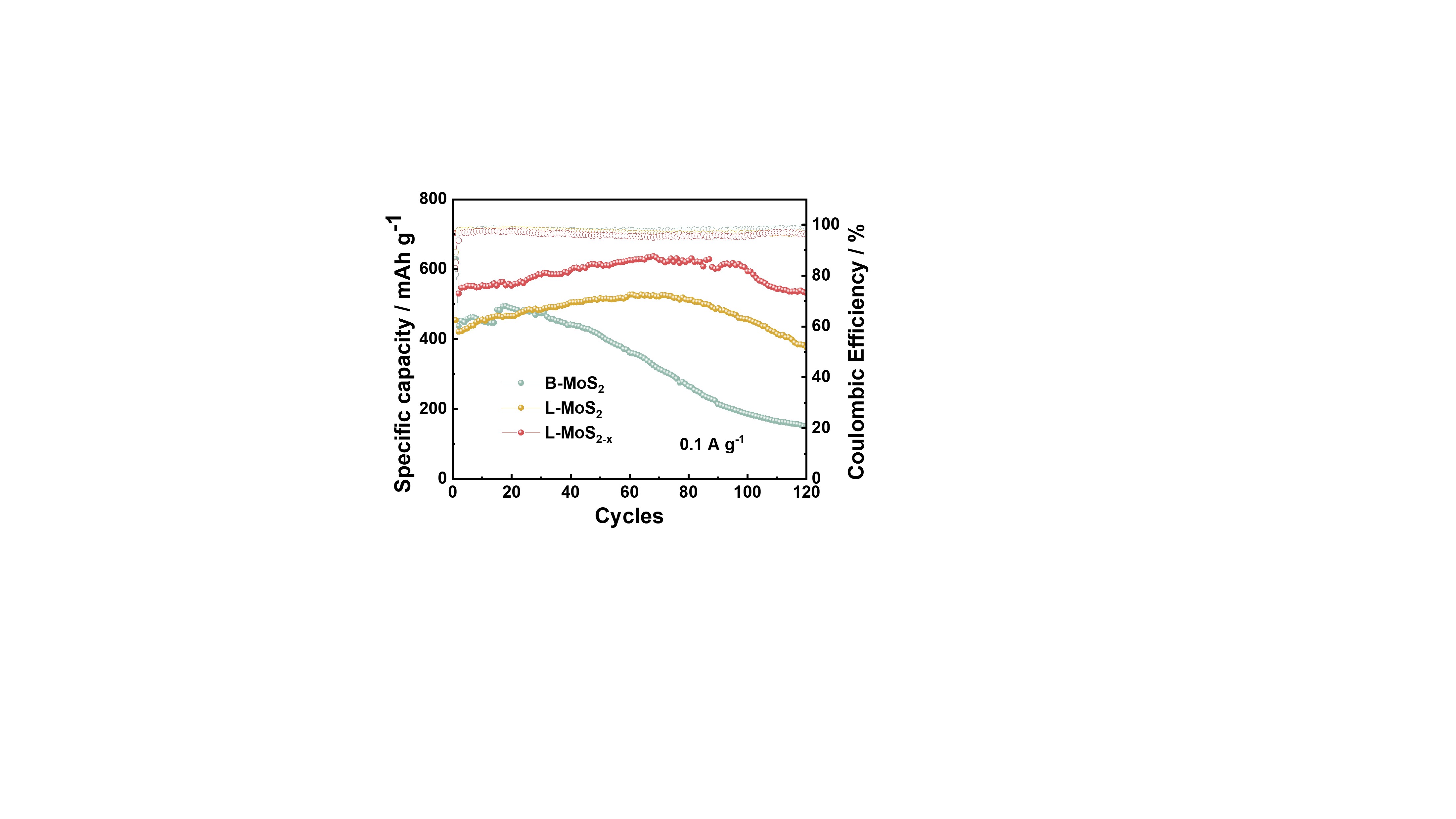


**Figure S19**. Cycling performances of MoS_2_, L-MoS_2_ and L-MoS_2-x_ at the current of 0.1 A g^-1^.


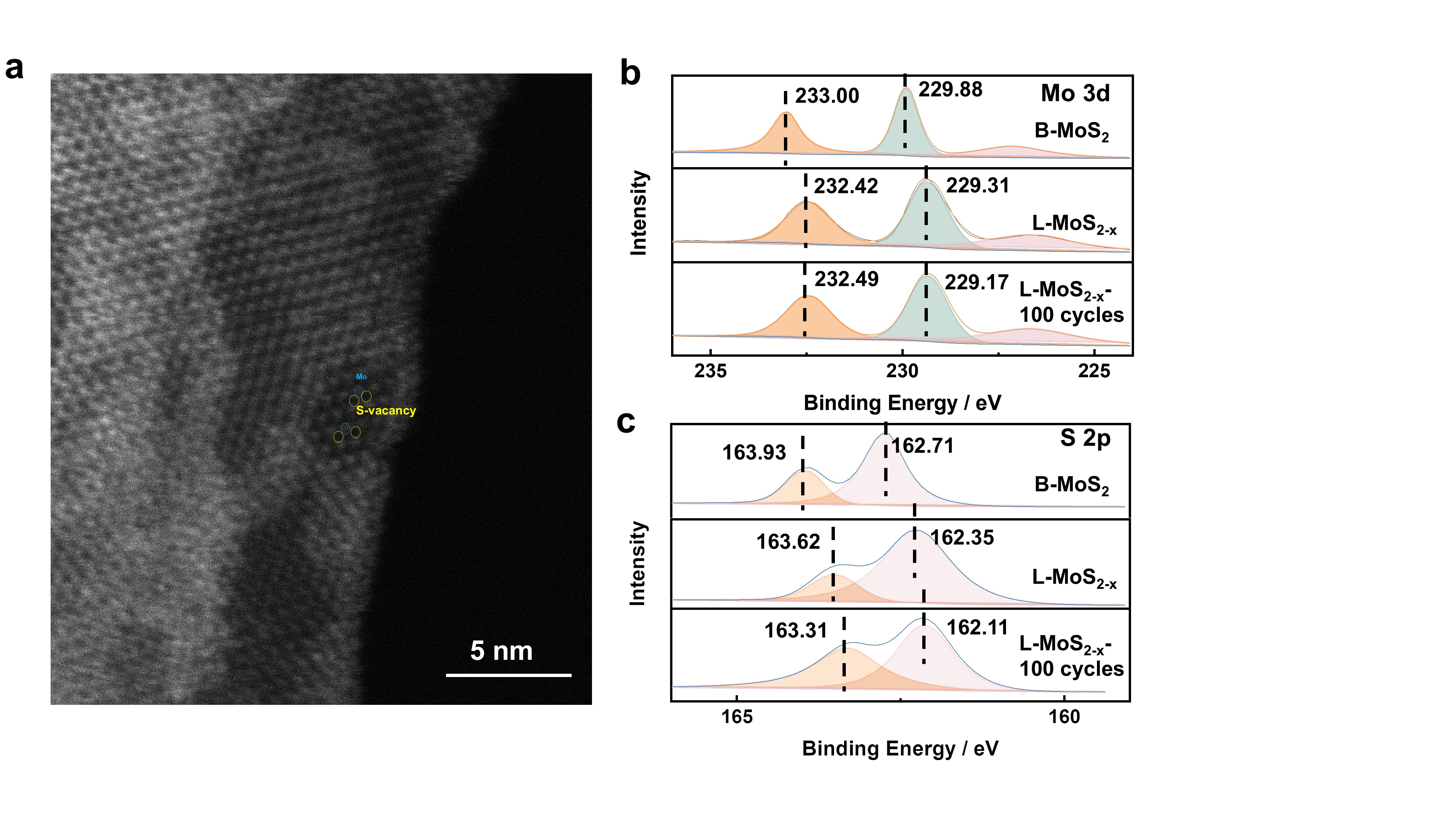


**Figure S20**. (a) The spherical aberration-corrected transmission electron microscopy (AC-TEM) of L-MoS_2-x_ after 100 cycles in sodium-ion batteries; (b) The Mo 3d XPS analysis of B-MoS_2_, L-MoS_2-x_ L-MoS_2-x_-100 cycles in sodium-ion batteries; c) The S 2p XPS analysis of B-MoS_2_, L-MoS_2-x_ L-MoS_2-x_-100 cycles in sodium-ion batteries;


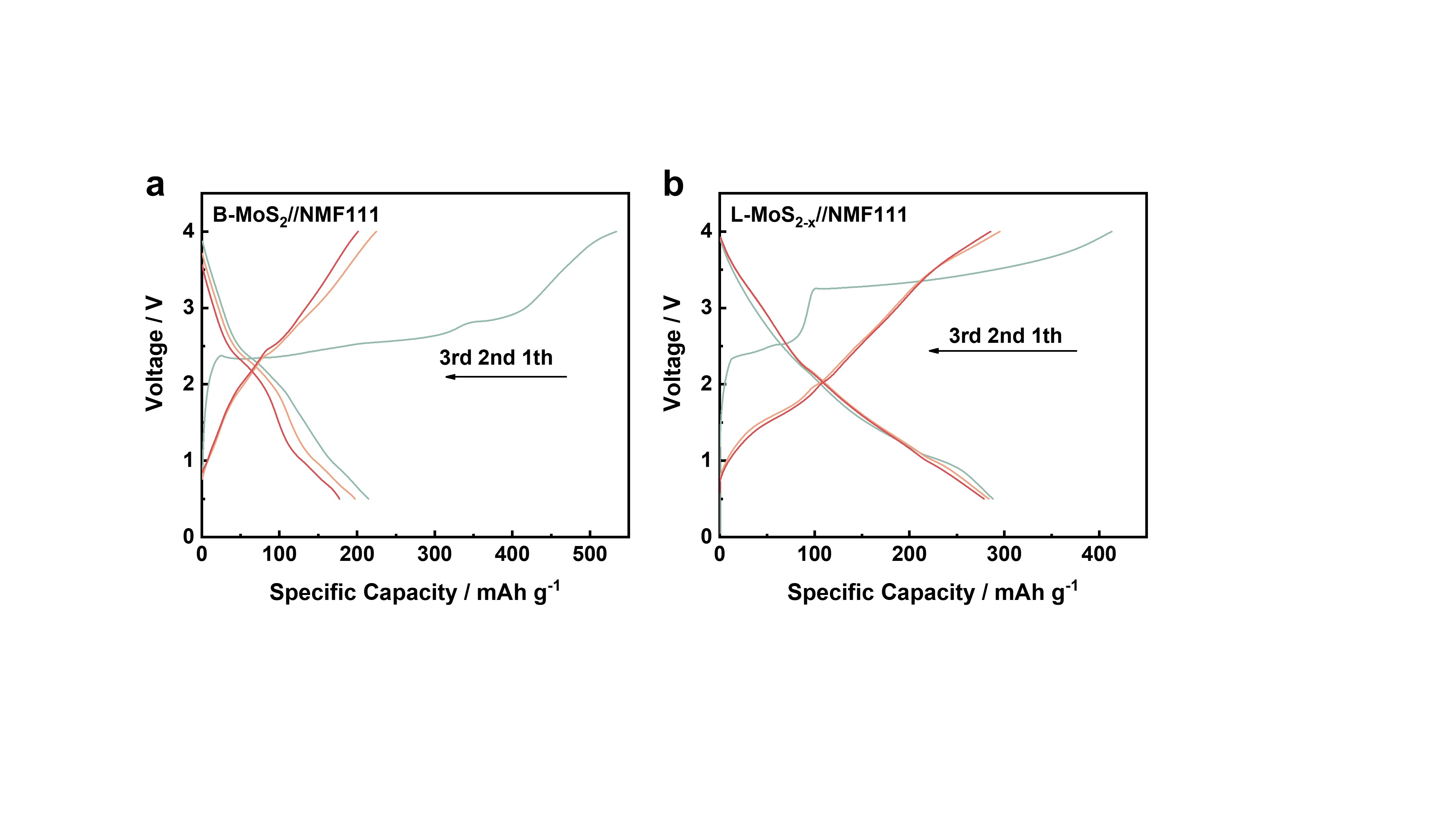


**Figure S21.** The galvanostatic charging/discharging curves of full cells for the first 3 cycles. (a) B-MoS_2_//NFM111. (b) L-MoS_2-x_//NFM111.


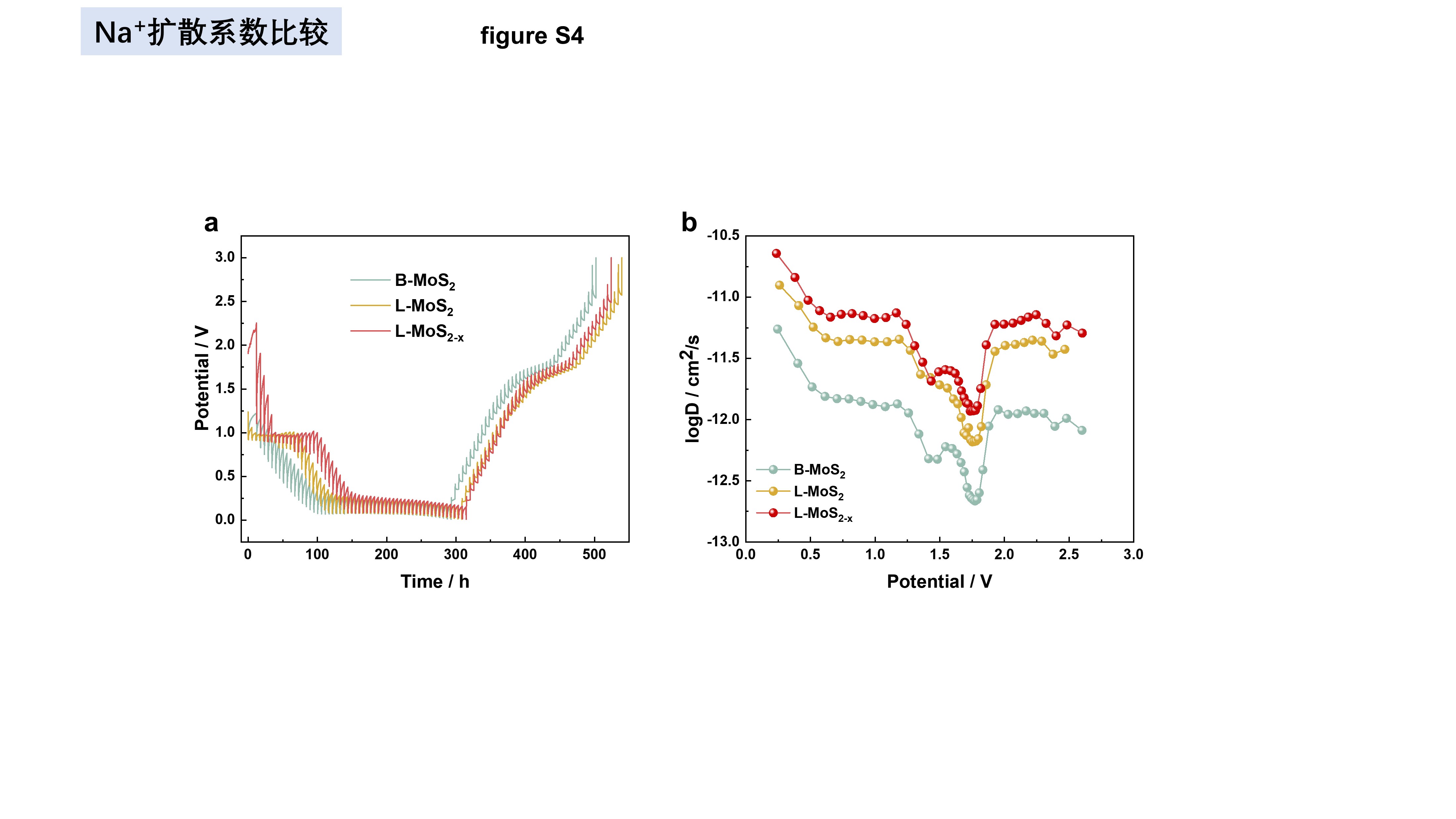


**Figure S22.** (a) The GITT test curves of L-MoS_2-x_ (b) Na^+^ chemical diffusion coefficients as a function of stoichiometry based on GITT data.


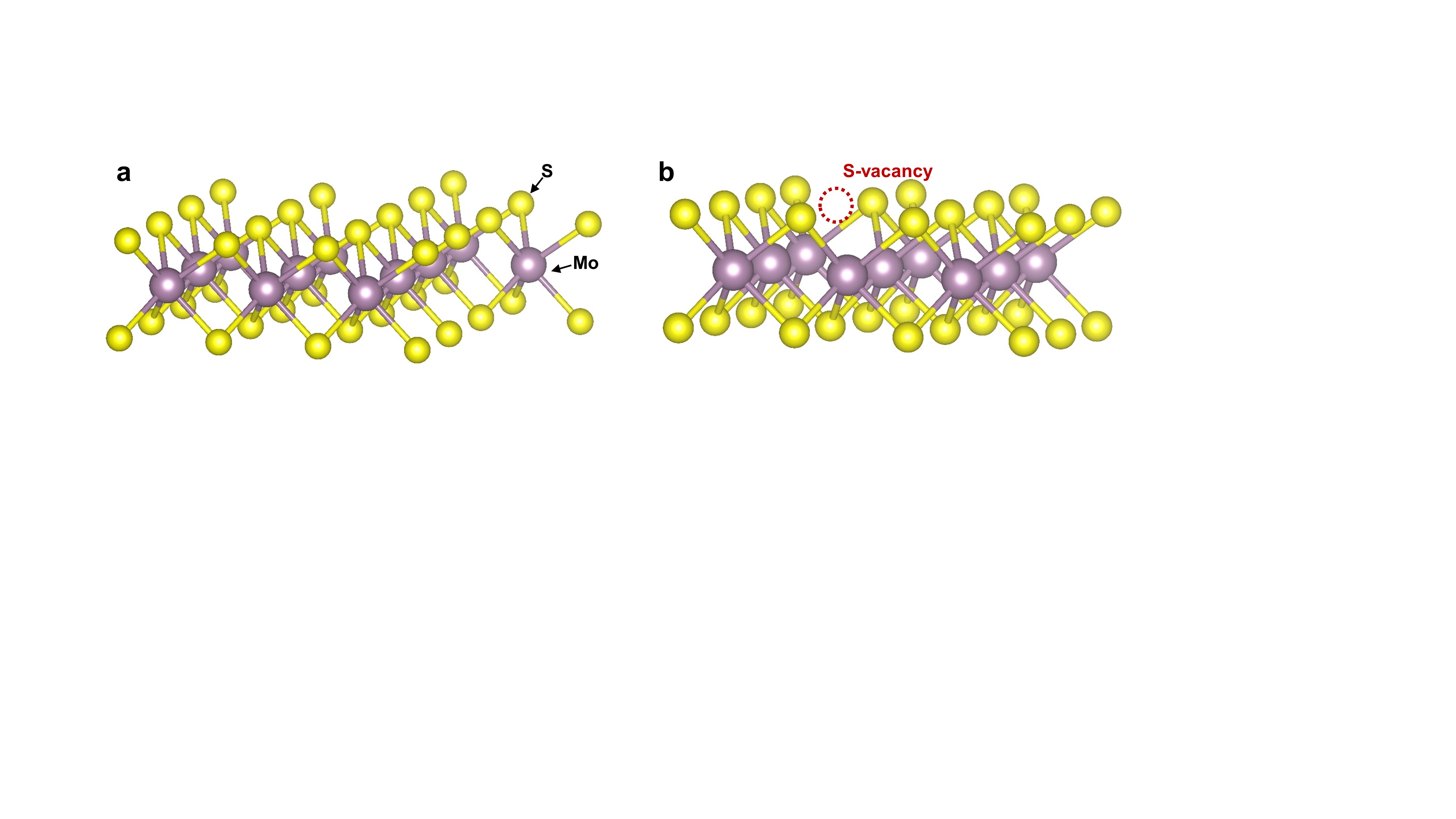


**Figure S23.** The DFT models of B-MoS_2_ and L-MoS_2-x_.


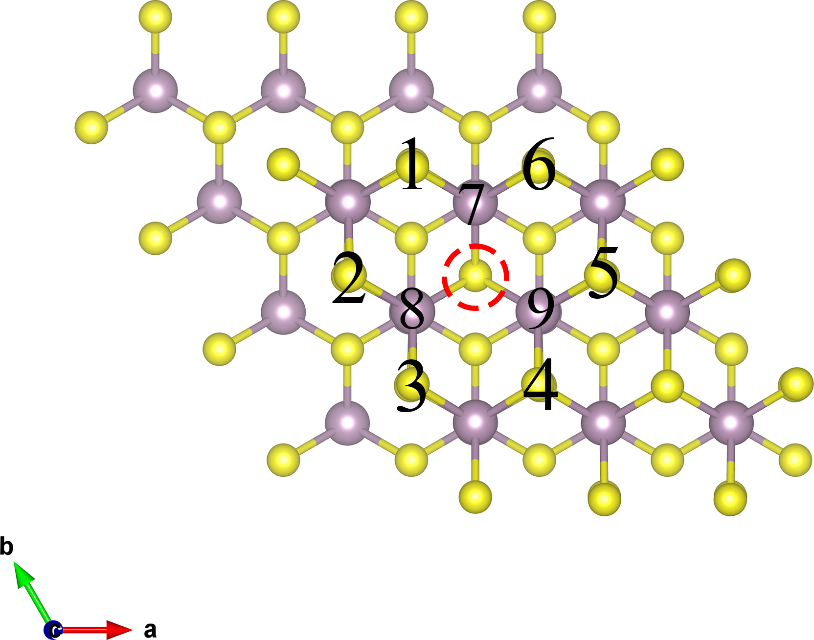


**Figure S24**. The illustration of the corresponding atoms in L-MoS_2-x_ and MoS_2_ for Bader charge analysis. The red circle indicates the S-vacancy in L-MoS_2-x_.


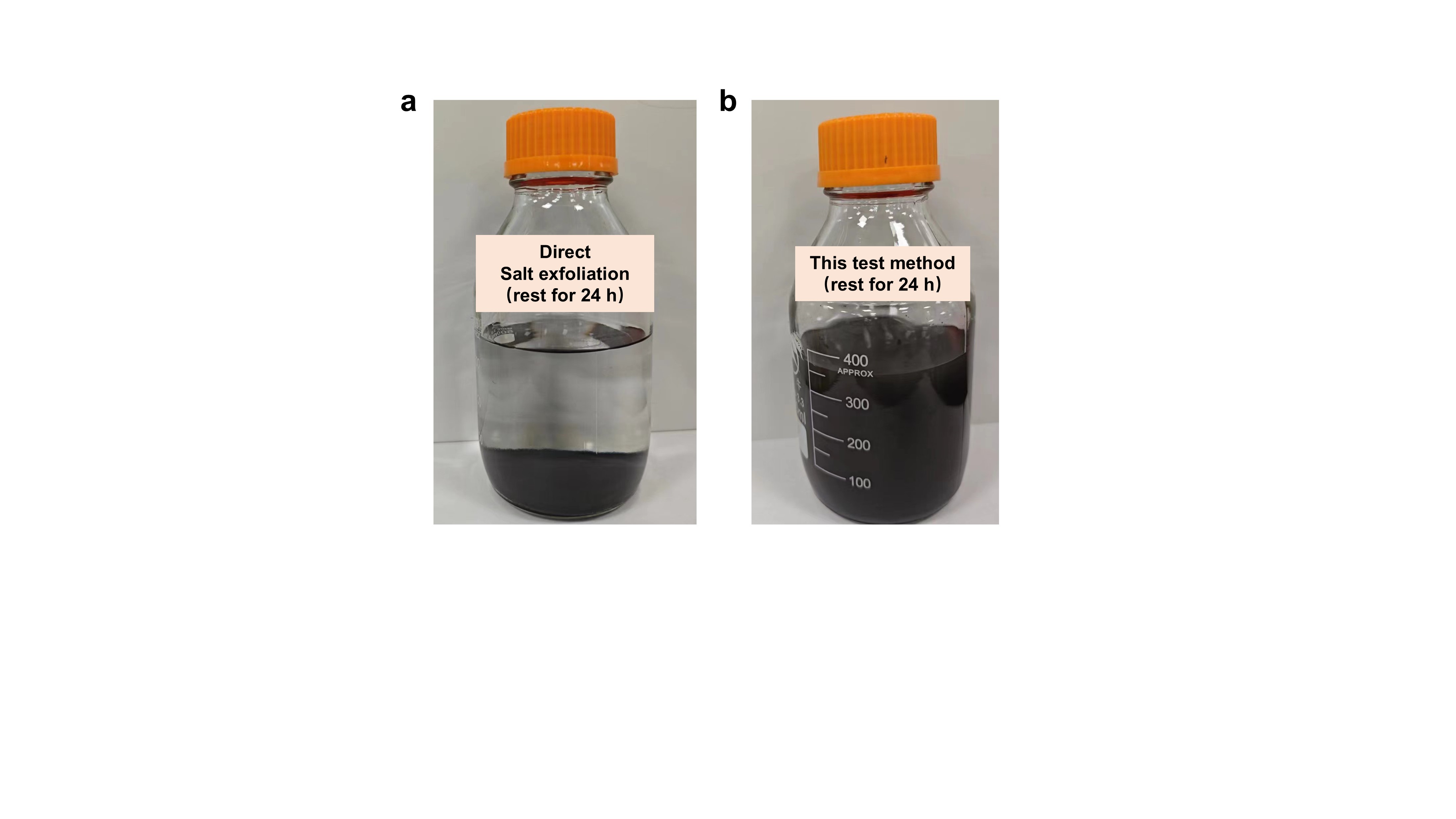


**Figure S25.** The state of exfoliation dispersions after 24 h of resting obtained by different exfoliation methods.

**Table S1** Bader charge of L-MoS_2-x_ and MoS_2_.

| Atom number shown in Figure S24 | Element | L-MoS_2-x_ | MoS_2_ |
| --- | --- | --- | --- |
| 1 | S1 | -0.49507 | -0.45471 |
| 2 | S2 | -0.49156 | -0.4539 |
| 3 | S3 | -0.49454 | -0.45381 |
| 4 | S4 | -0.49455 | -0.46107 |
| 5 | S5 | -0.49234 | -0.45549 |
| 6 | S6 | -0.49722 | -0.46171 |
| 7 | Mo1 | 0.824433 | 0.922141 |
| 8 | Mo2 | 0.847612 | 0.920123 |
| 9 | Mo3 | 0.826525 | 0.925868 |

**Reference**

[1] G. Kresse, J. Furthmüller, Computational Materials Science 1996, 6, 15-50.

[2] P. E. Blöchl, Physical Review B 1994, 50, 17953-17979.

[3] J. P. Perdew, W. Yue, Physical Review B 1986, 33, 8800-8802.

[4] S. Grimme, Journal of Computational Chemistry 2006, 27, 1787-1799.

[5] X. Wang, X. Shen, Z. Wang, R. Yu, L. Chen, ACS nano 2014, 8, 11, 11394–11400.

[6] Z. Zhu, Y. Tang, W. R. Leow, H. Xia, Z. Lv, J. Wei, X. Ge, S. Cao, Y. Zhang, W. Zhang, H. Zhang, S. Xi, Y. Du and X. Chen, Angew. Chem., Int. Ed. 2019, 58, 3521–3526.

[7]X. Du, X. Guo, J. Huang, Z. Lu, H. Tan, J. Huang, Y. Zhu and B. Zhang, Nanoscale Horiz. 2020, 5, 1618-1627.

[8]D. Xie, X. Xia, Y. Wang, D. Wang, Y. Zhong, W. Tang, X. Wang, J. TuNitrogen- Chem. A Eur. J., 22 (2016), pp. 11617-11623
